# Comprehensive analysis of annexin gene family and its expression in response to branching architecture and salt stress in crape myrtle

**DOI:** 10.1186/s12870-024-04748-8

**Published:** 2024-01-30

**Authors:** Hui Wei, Jinxin Chen, Xingyue Zhang, Zixuan Lu, Bilin Lian, Guoyuan Liu, Yanhong Chen, Fei Zhong, Chunmei Yu, Jian Zhang

**Affiliations:** 1 Key Laboratory of Landscape Plant Genetics and Breeding, School of Life Sciences, Nantong University, Nantong, 226001 China; 2 Key Lab of Landscape Plant Genetics and Breeding, Nantong, 226000 China

**Keywords:** Crape myrtle, Annexin, Branching architecture, Salt treatment

## Abstract

**Background:**

Annexin (ANN) is calcium (Ca^2+^)-dependent and phospholipid binding protein family, which is involved in plant growth and development and response to various stresses. However, little known about *ANN* genes were identified from crape myrtle, an ornamental horticultural plant widely cultivated in the world.

**Results:**

Here, 9 *LiANN* genes were identified from *Lagerstroemia indica*, and their characterizations and functions were investigated in *L. indica* for the first time. The *LiANN* genes were divided into 2 subfamilies. The gene structure, chromosomal location, and collinearity relationship were also explored. In addition, the GO annotation analysis of these *LiANNs* indicated that they are enriched in molecular functions, cellular components, and biological processes. Moreover, transcription factors (TFs) prediction analysis revealed that bHLH, MYB, NAC, and other TFs can interact with the *LiANN* promoters. Interestingly, the *LiANN2/4/6–9* were demonstrated to play critical roles in the branching architecture of crape myrtle. Furthermore, the *LiANN2/6/8/9* were differentially expressed under salt treatment, and a series of TFs regulating *LiANN2/6/8/9* expression were predicted to play essential roles in salt resistance.

**Conclusions:**

These results shed light on profile and function of the LiANN gene family, and lay a foundation for further studies of the *LiANN* genes.

**Supplementary Information:**

The online version contains supplementary material available at 10.1186/s12870-024-04748-8.

## Introduction

Annexin (ANN), a kind of calcium (Ca^2+^)-dependent and phospholipid-binding protein, is evolutionarily conserved and exists in eukaryotes and prokaryotes [1]. Plant ANNs are a multigene family and have been identified from Arabidopsis [2], rice [1], wheat [3], and poplar [4]. In view of significant function in Ca^2+^ signaling pathways, plant ANNs endowed with Ca^2+^ channel regulatory activity serve as important component of stress signal transduction [5]. Plant ANNs also have the peroxidase and ATPase/GTPase activities, which are involved in multiply regulatory effects on plant growth and development and stress resistance [6, 7]. The conserved repeats or domains of plant ANNs are characterized during the ANN family evolution. In general, plant ANNs contain a conserved C-terminal structure and a variable N-terminal sequence [8]. One or two repeats localized on C-terminus are identified as conserved domains in plant ANNs [8]. Previous studies suggested that the sequestration of Ca^2+^ at membrane interface depends on the degree of ANN conservation and oligomerization and has a distinguished influence on ANN-membrane interaction, ANN repeats possess, divergent lipid specificities, and unequal membrane-binding process [9, 10]. In addition, sequence alignment analysis reveals that a series of conserved motifs are identified in plant ANNs. For example, GXGT-(38 amino acids)-D/E found in repeats I and IV has a close association with Ca^2+^-dependent phospholipid binding activity [11]. An actin binding domain and a GTP binding domain (DXXG) are identified in repeats III and IV, respectively [12]. Moreover, plant ANNs contain a variable amino-terminal and C-terminal regions that are endowed with function of post-translational modifications, which are considered to be regulators for Ca^2+^-dependent signaling transduction [13]. For example, *Arabidopsis thaliana* ANN1 (AtANN1) modified by phosphorylation had the significant peroxidase activity in vitro, and AtANN1 after modification of S-glutathionylation resulted in promotion of Ca^2+^-binding activity [14, 15]. Besides the post-translational modifications, ANNs are also found to interact with functional proteins, including CDPK, MAPKK, TCTP, SYP, and actin. A *Hevea brasiliensis* ANN was identified to interact with HbTCTP, which played an essential in controlling metal-catalyzed oxidation system [16]. A recent report revealed that interaction of *Oryza sativa* ANN1 (OsANN1) and OsCDPK24 regulates H_2_O_2_ content and redox homeostasis in response to abiotic stress [17]. A *Gossypium hirsutum* ANN, GhFAnnxA, interacted with actin, which played important roles in fiber elongation and secondary cell wall biosynthesis [18].

The ANN property like Ca^2+^ and membrane binding is pivotal to their involvement in growth and development as well as response to various stresses. Overexpression of cotton *GhAnn3* in Arabidopsis significantly changed the trichome density and leaf length [19]. Silencing *GhAnn2* expression had a distinguished influence on Ca^2+^ implantation at the cell apex, which inhibited cotton fiber elongation [20]. Arabidopsis *AtAnn5*, specifically expressed in mature pollen, had been identified to be involved in both pollen generation and development and pollen tube growth [21]. In *Arachis hypogaea*, the expression levels of *AhANN1*, *5*, *6* were upregulated under NaCl treatment, and *AhANN3* mRNA was significantly improved by cold treatment [22]. In soybean, the transcript levels of *GmANN1*, *11*, and *12* were accumulated under salt, drought, and abscisic acid (ABA) stresses [23]. In *Triticum aestivum*, *TaANN10* was speculated to be involved in the cold-induced male sterility. The *TaAnn12-A/D* expression was identified to accumulate under drought and cold stresses, and the transcript level of *TaAnn2* was significantly improved under NaCl stress [3]. In Arabidopsis, AtANN1 and AtANN4 were involved in regulating resistance to salt and drought stresses by the Ca^2+^-dependent signal transduction [24]. Also, the transcript level of *AtANN1* was promoted by heat stress and AtANN1 positively mediated heat tolerance in Arabidopsis by increasing in [Ca^2+^]_cyt_ [25]. The *OsANN1* from rice was induced by high temperature and overexpression of *OsANN1* dominantly enhanced heat tolerance in rice by regulating ROS production [17]. Overexpression of *OsANN3* improved stomatal closure and ABA accumulation in rice cells, which promoted tolerance to ABA and drought stresses [12]. In addition, overexpression of *BjANN2* improved salt tolerance by regulating proline accumulation and maintaining ion homeostasis [26]. The *Zea mays* ANN33/35 (ZmANN33/35) had been identified to be associated with cold stress, and accumulation of *ZmANN33*/*35* expression levels led to rapid recovery from the cell membrane damage [27]. Taken together, the studies indicated that ANNs possess significant function in plant morphogenesis, including regeneration, growth, and development. Plant ANNs endowed with various kinds of physiological function play essential roles in both plant growth and response to environmental stresses.

Crape myrtle (*Lagerstroemia indica*) planted worldwide, a member of *Lythraceae* family, is an important horticultural plant [28]. *L. indica* exhibiting attractive plant architectures is cultivated extensively as ornamental species along streets and roadsides. The *L. indica* architecture considered as an important evaluation index is significantly affected by environmental and genetic factors [29]. It was reported that plant ANNs are involved in plant growth and multiple morphogenesis [30], however, the characterizations and functions of crape myrtle ANNs in regulatory mechanism of branching architecture are still unavailable. Additionally, previous reports displayed that ANNs are involved in various stresses, including salt stress. However, whether the crape myrtle ANNs respond to salt stress is still an unresolved issue. In this study, nine *LiANN* genes were first identified from *L. indica* and clustered into two subfamilies. Besides, the conserved motifs, gene structures, and putative phosphorylation sites of the *LiANN* genes were systematically analyzed, and the chromosomal localizations and syntenic gene pairs were investigated based on the genome database. The transcript profiles of the *LiANN* genes during different stages of branching architecture and under salt treatment were identified. In summary, these results provided critical insights into the molecular functions of the *LiANN* genes on *L. indica* architecture and response to salt stress.

## Materials and methods

### Genome-wide investigation of the *LiANNs*

We could obtain the genomes of *A. thaliana*, *O. sativa*, *Eucalyptus grandis*, *Vitis vinifera*, *Populus trichocarpa*, and *Salix purpurea* from Phytozome (http://www.phytozome.net/). To identify the crape myrtle *ANN* genes, we retrieved the HMM model PF00191 of ANN domain from the Pfam database (https://pfam.xfam.org) and searched the *L. indica* genome databases with an e-value cutoff of 1.0 × e^−10^ using TBtools software. In addition, we used the AtANN protein sequences, including AtANN1 (AT1G35720), AtANN2 (AT5G65020), AtANN3 (AT2G38760), AtANN4 (AT2G38750), AtANN5 (AT1G68090), AtANN6 (AT5G10220), AtANN7 (AT5G10230), and AtANN8 (AT5G12380), to blast against the local protein databases of *L. indica* with an e-value cutoff of 1.0 × e^−10^ and identities > 40%. To further confirm the LiANN candidates, we used the SMART, Pfam and CDD database to ensure the completeness of conserved domains and motifs. We applied the Cell-PLoc 2.0 program to predict the subcellular localizations of the LiANNs. Also, we used the ExPASy to calculate molecular weights (MWs), isoelectric points (pI), and grand averages of hydropathicity (GRAVYs), and evaluate the instability of the LiANNs.

### Multiple alignment and phylogenetic analysis

We used ClustalW to generate the multiple alignments of the LiANNs, and analyzed the conserved domains and speculated the putative function. In addition, we investigated the phylogenetic relationship of the ANNs among *L. indica*, *A. thaliana*, *O. sativa* and *P. trichocarpa*. The ANN protein sequences of Arabidopsis, rice and poplar have been identified from the previous studies [1, 4]. We used the MEGA7.0 software with: p-distance, pairwise deletion, and 1000 bootstrap replicates to generate the phylogenetic tree and applied the iTOL (https://itol.embl.de/) to visualize the phylogenetic tree [31, 32].

### Chromosomal localization, gene duplication, and molecular evolution

For chromosomal localizations of the *LiANNs*, we retrieved the locus information of the *LiANNs* and fixed them on the corresponding chromosomes based on the gff3 annotation file. In addition, we applied Multiple Collinearity Scan toolkit (MCScanX) to identify the syntenic relationship among the *LiANN* members. To further understand the role of gene pairs in evolutionary process, we used MCScanX to identify the putative syntenic gene pairs among *L. indica*, *A. thaliana*, *O. sativa*, *Eucalyptus grandis*, *V. vinifera*, *P. trichocarpa*, and *S. purpurea*. To explore the signatures of selection pressure on the *LiANN* evolutionary process, we extracted the coding sequences (CDSs) of the *LiANN* gene pairs and calculated the values of Ka (nonsynonymous) and Ks (synonymous).

### Gene structure and protein motif detection

We used the *L. indica* genome database and gff3 annotation file to investigate the exon and intron distributions of the *LiANN* genes. To identify the influence of exon and intron on *ANN* evolution, we also analyzed the exon and intron locations of the *AtANN*, *OsANN*, and *PtANN* genes using the similar methods. In addition, we applied the Multiple Expectation Maximization for Motif (MEME) online tool to identify conserved motifs of the ANN proteins. Moreover, we used the TBtools to visualize the phylogenetic tree, motif compositions, and gene structures [33]. In general, protein structure has a close association with protein function, and the structural integrity contributes to performance of protein function during the physiological process. We applied the SWISS-MODEL and Chimera software to identify and compare the structures of the LiANN proteins.

### *Cis*-element, transcription factor (TF), interaction network and gene ontology (GO) analysis

To provide insight into *cis*-regulatory model of the *ANN* genes across different species, we extracted the 2 kb upstream regions of the *AtANN*, *OsANN*, *PtANN*, and *LiANN* genes and submitted them to PlantCARE [34]. We used the TBtools to integrate and visualize the *cis*-acting elements of the *ANN* promoters. To investigate the putative TFs which can interact with the specific sites of the *LiANN* genes, we submitted the *LiANN* promoter sequences to the PlantRegMap database which contains a series of TF regulatory information. In addition, based on homologous proteins of the LiANNs in Arabidopsis, we used the String database and Cytoscape software to predict and visualize the interaction network [35].

### RNA-sequencing and qRT-PCR analysis

As an ornamental plant, the branching architecture of *L. indica* is one of the plausible traits. Different *L. indica* varieties with various angels have been reported, but the regulation mechanism of branch development has not been elucidated. From the *L. indica* germplasms, we found a variety endowed with horizontal branch and the variety was named as the “Li18-4”. The variety has no apical dominance, and most of branches grow almost in horizontal manner, which contributed to elucidating branching mechanism under same genomic background. To evaluate the putative regulation mechanism of branching architecture, we treated the grow state of the “Li18-4” as follows: (1) The samples in the first group (group A/B) maintained normal growth; (2) The samples in the second group (group C/D) were rotate by 180°; (3) The samples in the third group (group E/F) were rotate by 90°; and (4) the samples in the fourth group (group G/H) were rotate by 270°. The A, C, E, and G indicated the upper tissues of the bending sites (UTBS), while the B, D, F and H represented the lower tissues of the bending sites (LTBS). (Supplemental Fig. 1). Branches or buds were cut from the “Li18-4” variety and performed for RNA-sequencing (RNA-seq) (CNP0003990). Subsequently, the RNA was extracted using the Plant RNA Extraction Kit (TIAN GEN) and reverse transcribed into first strand cDNA using the PrimeScript™ RT Master Mix (TaKara). The ABI 7500 Fast Real-Time PCR System (Thermo Fisher Scientific) was applied to illustrate the *LiANN* expression patterns using the UltraSYBR Green I Mixture (CWBIO). Also, *LiActin* (*evm.model.Chr10.488*) considered as the internal control was used to calculate the relative expression levels of the *LiANNs* based on 2^−ΔΔCT^ method. The primers for qRT-PCR were shown in Supplemental Tab. 1. Additionally, the log2 method and TBtools were applied to normalize and visualize the relative expression levels of the *LiANNs*, respectively. To investigate the putative mechanism of the *L. indica* under salt stress, we classified the *L. indica* germplasms into salt sensitive and salt tolerant varieties. The salt sensitive and salt tolerant varieties were cultivated the same region, and the 200 mM NaCl was used to irrigate the *L. indica*. Plant samples for RNA-seq were immediately frozen in liquid nitrogen and the corresponding RNA was extracted for RNA-seq (CNP0003991). Subsequently, to deeply understand the putative functions of the LiANNs under the salt stress, The semi-hardwood *L. indica* grown into mixed soil were irrigated with water containing 200 mmol/L NaCl. The adventitious roots were collected at 0 (control), 1, 3, and 5 d of NaCl treatment, and RNA was extracted as well as reverse transcribed. The qRT-PCR was also used to illustrate the relative expression levels of the *LiANNs* under the NaCl treatment.

**Fig. 1:**
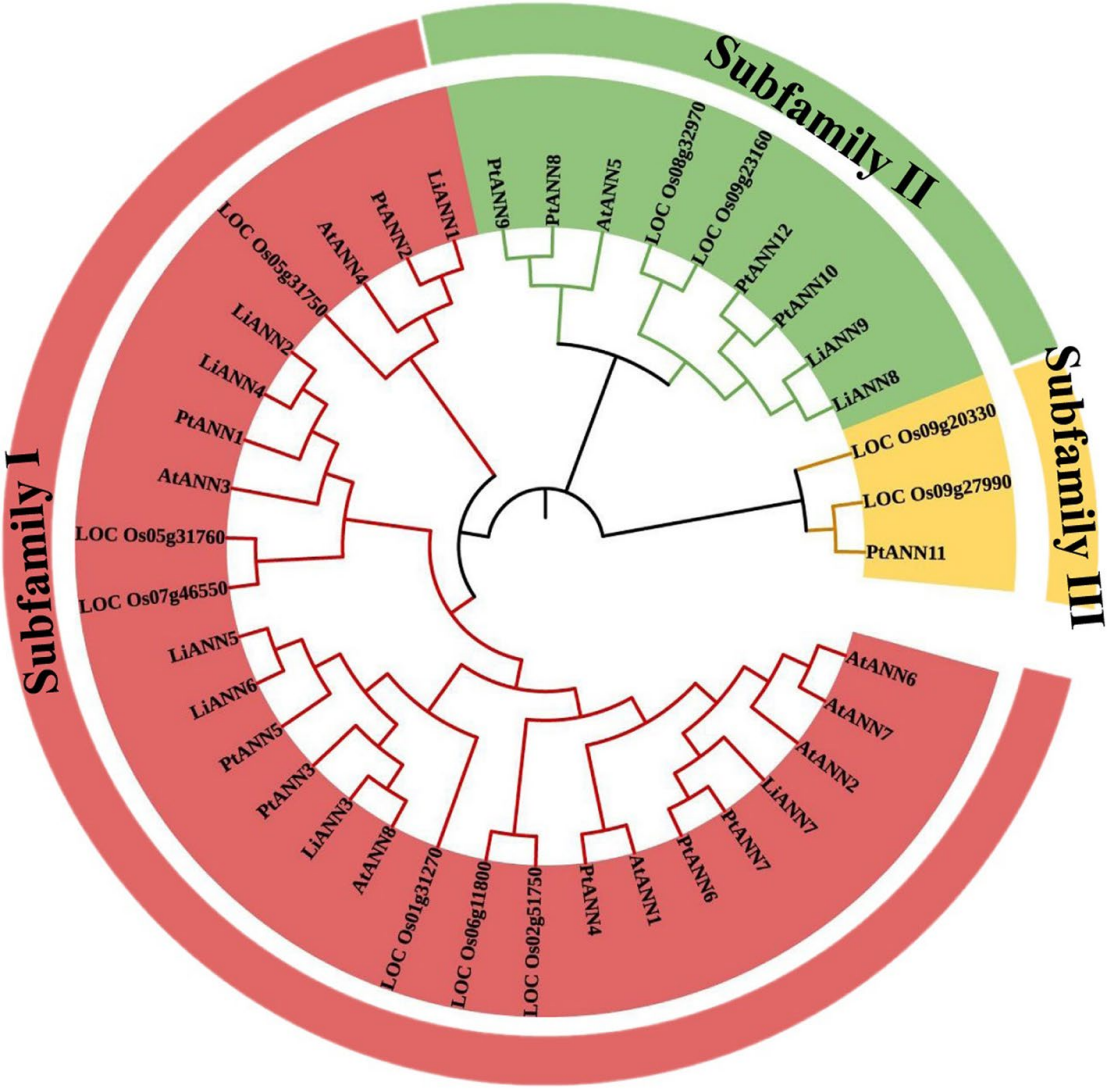
Phylogenetic tree of ANN families in *Lagerstroemia indica*, *Arabidopsis thaliana*, *Oryza sativa* and *Populus trichocarpa*. The different colors indicated different subfamilies of ANNs. The full-length ANN sequences were performed to construct neighbor-joining phylogenetic tree using MEGA7

## Results

### Genome-wide identification of the ANN family genes in *L. indica*

To identify the *L. indica* ANN family, we performed a pfam (PF00191) search of *L. indica* genome database, and a total of 11 *LiANN* candidates were retrieved from the *L. indica* genome (Supplemental Tab. 2). Also, we applied a BLASTP search of *L. indica* genome database using 8 AtANN and 10 OsANN sequences, and 9 LiANNs with high sequence similarity were identified from the *L. indica* (Supplemental Tab. 2). Taken together, a total of 9 *ANN* gene sequences (including *evm.model.Chr5.9.2*, *evm.model.Chr5.10*, *evm.model.Chr8.396*, *evm.model.Chr9.51*, *evm.model.Chr10.925*, *evm.model.Chr10.927*, *evm.model.Chr11.901*, *evm.model.Chr12.256*, and *evm.model.Chr16.853*) were identified from *L. indica*. Based on the genomic position, these genes were named *LiANN1-9* (Supplemental Tab. 3). The length of the LiANN proteins ranged from 182 (LiANN5) and 347 (LiANN1) amino acids (aa) and most of LiANNs had lengths of 300 aa. The pI varied between 5.89 (LiANN2) and 9.18 (LiANN9). For MWs, the smallest was 20.87 kDa (LiANN5) and the largest was 39.96 kDa (LiANN1). The instability index ranged from 34.91 (LiANN9) to 61.04 (LiANN5), while LiANN2, 7, and 9 were considered as stable proteins (Supplemental Tab. 4). In addition, the GRAVY values of LiANN proteins were predicted to be negative, which suggested all LiANNs possess hydrophilic characteristics (Supplemental Tab. 4). Based on the results of subcellular localizations, the LiANNs were speculated to play a crucial role in cytoplasm (Supplemental Tab. 4).

### Conserved domains and phylogenetic tree of the LiANNs

In order to investigate the functions of the LiANN proteins, we used MEGA7.0 to identify 9 LiANN proteins, and highly conserved domains were detected (Supplemental Fig. 2). Similarity with other plant ANNs, LiANN proteins contained I, II, III, and IV repeats and several conserved domains. For example, type II Ca^2+^-binding sites (G-X-G-T-(38)-D/E) were found in the repeat I, but not in the repeats II-IV. Besides the conserved Ca^2+^-binding sites, there were 5 LiANNs (LiANN1, 3, 6, 7, and 9) containing conserved IRI motif in repeat III and 4 LiANNs (LiANN2, 4, 6, and 7) containing conserved DXXG motif in repeat IV. In addition, 3-dimensional (3D) structure prediction revealed that except for LiANN1 containing a small part of strand, LiANN proteins are composed of coils and helixes (Supplemental Fig. 3). Helixes accounted for a most of the LiANN structures, whereas coils consisted of a small of the LiANN structures. According to comparison of the LiANN structures, we found that LiANN proteins shared similar 3D structures. All these observations implied that structures and functions of the LiANNs are relatively conservative and relevant.

**Fig. 2:**
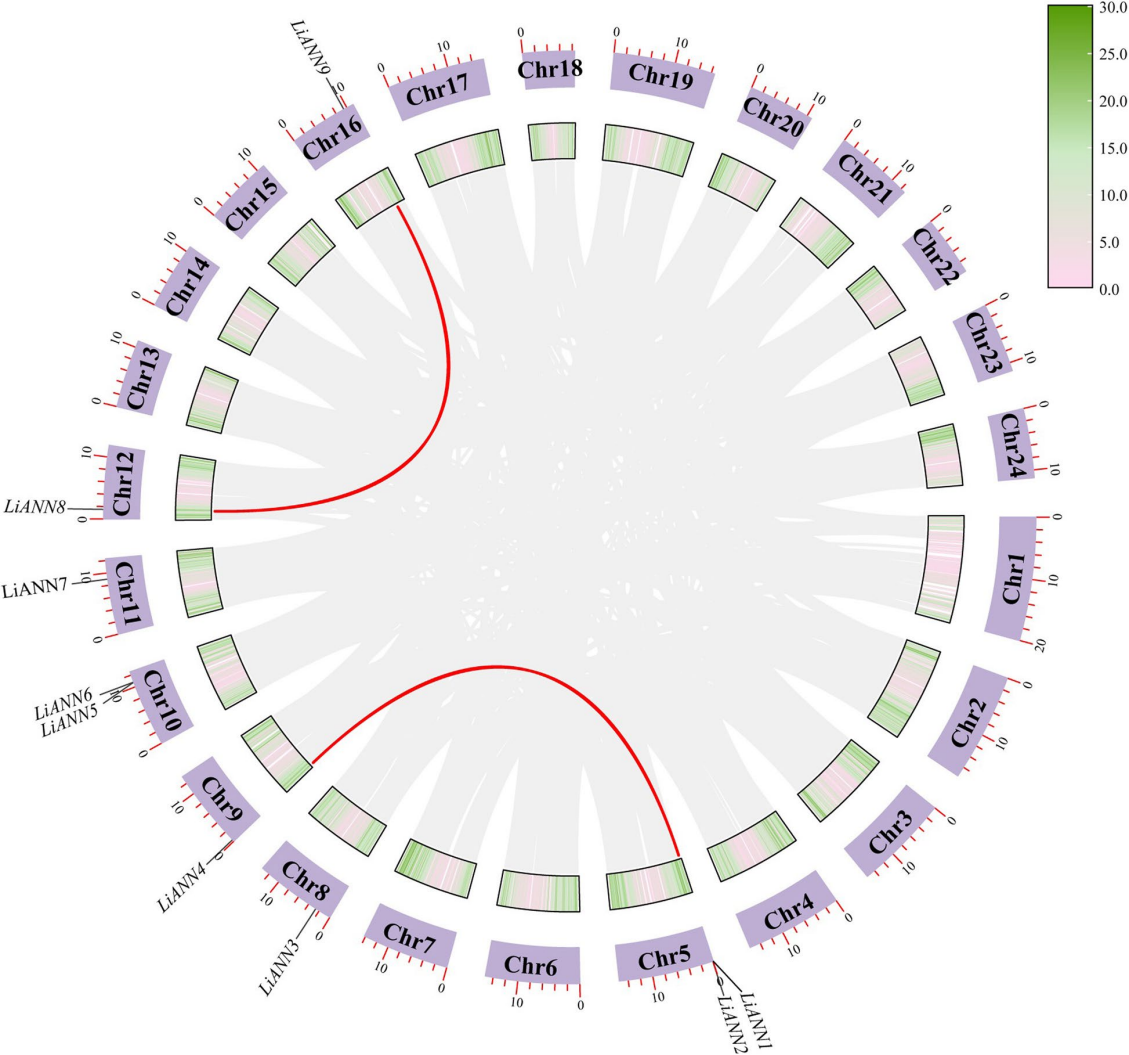
Synteny analysis of the *LiANN* genes in *L. indica*. Chromosomes 01–24 were represented by lilac rectangles. The gray lines represented gene pairs of *L. indica*

**Fig. 3:**
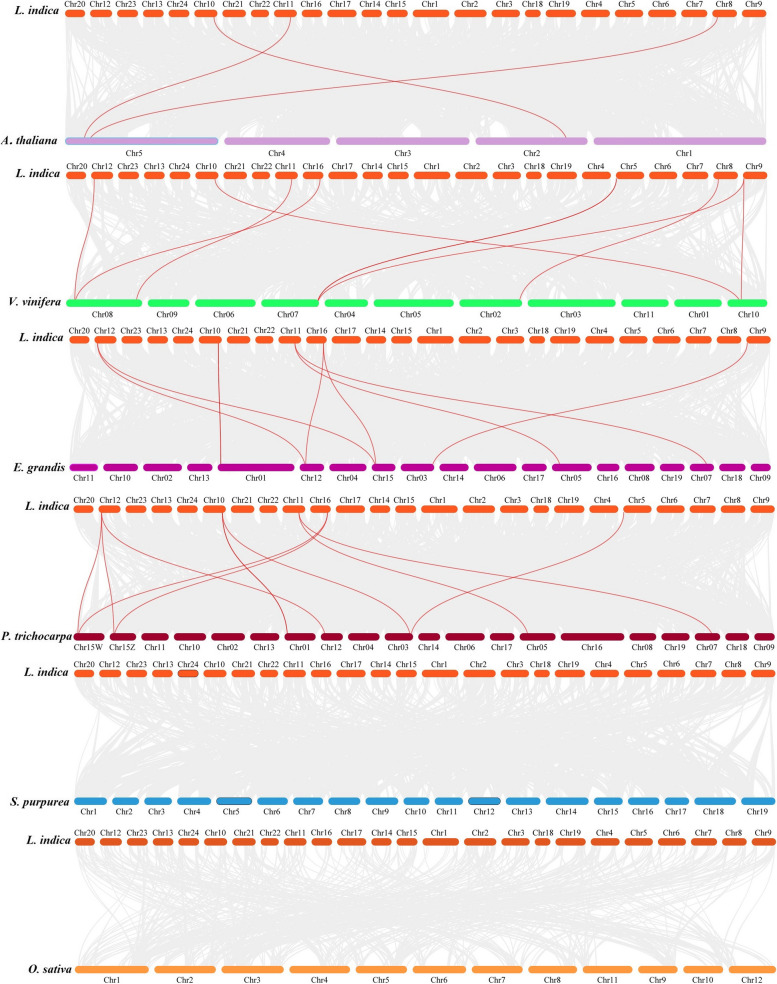
Syntenic relationship analysis of *ANN* genes among *L. indica*, *A. thaliana*, *O. sativa*, and *P. trichocarpa*, *Eucalyptus grandis*, *Vitis vinifera*, and *Salix purpurea*. Gray lines represented the collinearity gene pairs among *L. indica*, *A. thaliana*, *O. sativa*, and *P. trichocarpa*, *E. grandis*, *V. vinifera*, and *S. purpurea*, and the red lines indicated the *ANN* gene pairs

To explore the evolutionary relationship among ANN from different species, 9 *LiANNs*, 8 *AtANNs*, 10 *OsANNs*, and 12 *PtANNs* was performed to construct a phylogenetic tree (Fig. 1). All ANNs could be clustered into three subfamilies, the subfamily I contained 27 members (with 7, 7, 6, and 7 members of Arabidopsis, crape myrtle, rice, and poplar, respectively), the subfamily II had 9 members, and the subfamily III was composed of 3 members. Only 2 rice ANNs and 1 poplar ANN were clustered into subfamily III, which indicated ANN members of subfamily III are unique during the evolutionary process. In addition, out of nine LiANNs, the LiANN7 was present on the same clade with AtANN2/6/7; the LiANN3/5/6 showed similarity with AtANN8; the LiANN2/4 and AtANN3 were clustered into the same clade; the LiANN1 was grouped with AtANN4; and the LiANN8/9 showed high similarity with AtANN5.

### Phosphorylation site evaluation

We applied the NetPhosK 3.0 Server to investigate the post-translational modification of the LiANN phosphorylation sites, and the result suggested that LiANNs are composed of various phosphorylation sites (Supplemental Tab. 5). The serine, threonine, and tyrosine as the main phosphorylation sites were detected in the LiANNs. Among of them, serine was identified as the major phosphorylation sites, followed by threonine and tyrosine. For phosphorylation sites of the LiANN1, we found 9 kinds of kinase phosphorylation mode including PKA, ATM, cdc2, CKI, CKII, PKC, PKG, RSK, and unsp, while the LiANN8 contained 15 kinds of kinase phosphorylation mode, namely PKC, PKA, cdc2, unsp, cdk5, p38MAPK, GSK3, DNAPK, ATM, EGFR, INSR, RSK, CKI, CKII, and PKG. Additionally, the phosphorylation modes of all LiANNs were likely unsp, and PKC and PKA appeared the higher frequency of phosphorylation in most of the LiANNs.

### Chromosomal locations and syntenic relationship of the *LiANNs*

In order to determine the chromosomal distributions of the *LiANN* genes, the genomic locations of the *LiANN* genes were searched according to annotation file. The result indicated that 9 *LiANN* genes were distributed to 7 chromosomes, with no dominant correlation to chromosome length. (Supplemental Fig. 4). In total of 2 *LiANN* genes (*LiANN1/2*) were present on chromosome 5; 2 *LiANN* genes (*LiANN5/6*) were mapped on chromosome 10; and chromosome 8, 9, 11, 12, and 16 contained 1 *LiANN* gene, respectively.

**Fig. 4:**
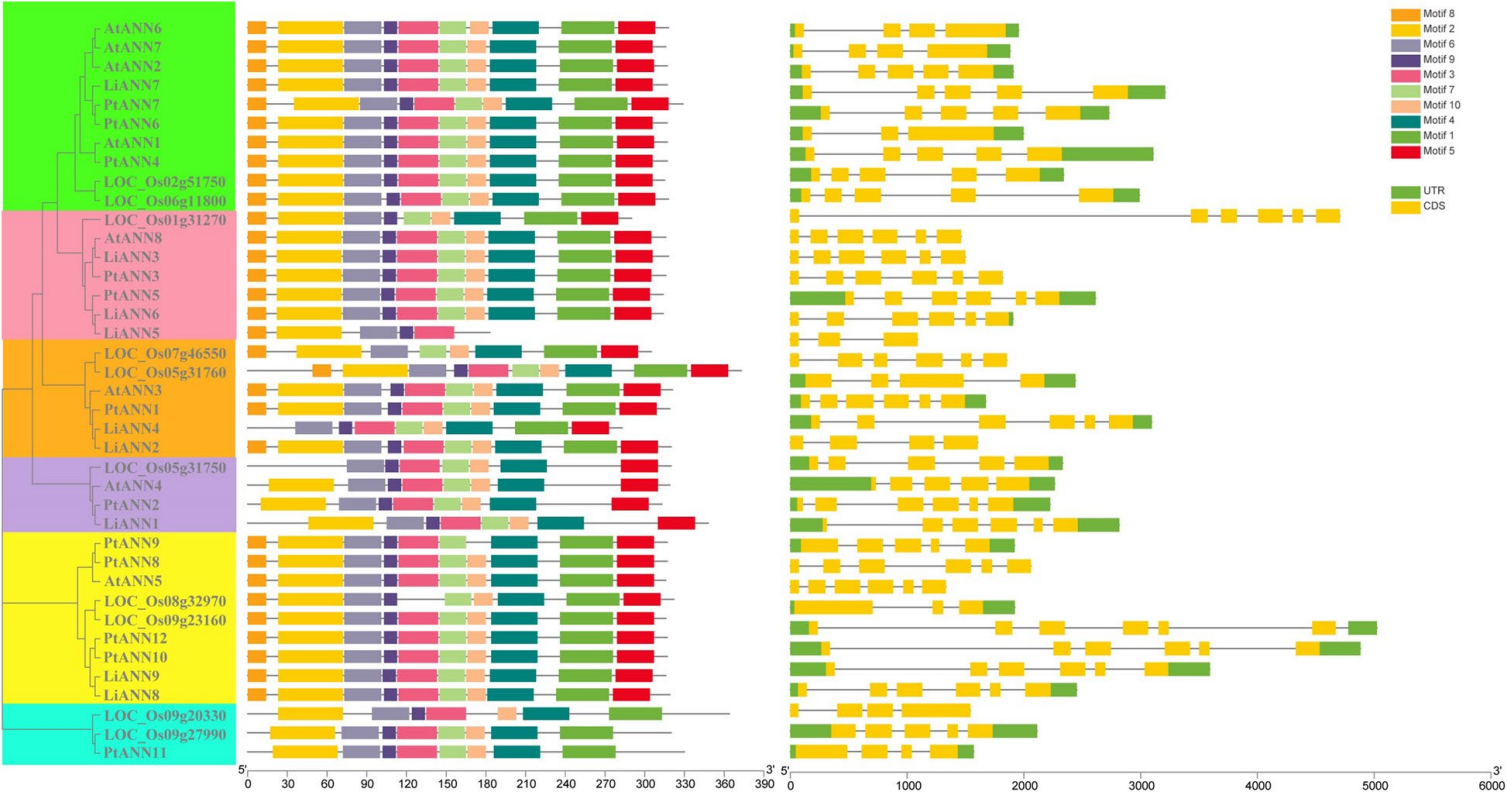
Architecture of conserved motifs and gene structures of *ANNs* from *L. indica*, *A. thaliana*, *O. sativa*, and *P. trichocarpa*

Gene duplication happens in plant evolutionary process, and tandem and segmental duplication events lead to gene expansion [36]. In order to further identify the diversity and evolution of the *ANNs* in *L. indica*, we examined gene duplication events in the *LiANNs*. A total of 3 duplicated *LiANN* gene pairs were determined in *L. indica*. Of them, 2 gene pairs of the *LiANN* genes (*LiANN2*/*LiANN4* and *LiANN8*/*LiANN9*) were identified as segmental duplication events ( Fig. 2), and 1 gene pair (*LiANN1*/*LiANN2*) was investigated as tandem duplication event. These results suggested that tandem and segmental duplication events play an essential role in evolutionary processes of the *LiANN* members and segment duplication events may be the main driving forces of the *LiANN* gene evolution. According to the syntenic *LiANN* gene pairs, the ratio of non-synonymous (Ka) and synonymous substitution (Ks) were calculated. In general, Ka/Ks ratio > 1 means gene under positive selection, Ka/Ks = 1 represents gene under the neutral selection, while Ka/Ks < 1 suggests gene under negative selection [37]. The Ka/Ks of syntenic *LiANN* gene pairs was less than 1, which indicated that the *LiANNs* may have experienced purifying selective pressure during the evolutionary process.

To further identify the evolutionary relationship of the *ANNs*, we constructed a syntenic relationship of *L. indica* and other species including *A. thaliana*, *O. sativa*, *E. grandis*, *V. vinifera*, *P. trichocarpa*, and *S. purpurea* (Fig. 3). Collinearity results showed that 3 syntenic orthologous gene pairs (*LiANN3*/*AtANN8*, *LiANN5*/*AtANN3*, and *LiANN7*/*AtANN6*) were identified between *L. indica* and *A. thaliana*, and 9 collinearity gene pairs (*LiANN4*/*PtANN5*, *LiANN5*/*PtANN1*, *LiANN6*/*PtANN2*, *LiANN7*/*PtANN6*, *LiANN7*/*PtANN7*, *LiANN8*/*PtANN10*, *LiANN8*/*PtANN12*, *LiANN9*/*PtANN10*, and *LiANN9*/*PtANN12*) were determined between *L. indica* and *P. trichocarpa*. In addition, a total of 9 or 11 syntenic *ANN* gene pairs were found between *L. indica* and *S. purpurea* or *E. grandis*, and no *ANN* gene pair was determined between *L. indica* and *O. sativa* or *V. vinifera* (Fig. 3). Interestingly, some *ANNs* between *L. indica* and *P. trichocarpa* or *S. purpurea*, such as *LiANN7-9*, had at least two syntenic gene pairs, which indicated that they may play crucial roles in the evolutionary processes of the *ANN* genes. Moreover, collinearity analysis revealed a higher homology among *L. indica*, *A. thaliana*, *E. grandis*, *P. trichocarpa*, and *S. purpurea* (dicot) than that between *L. indica* and *O. sativa* (monocot). Compared with monocots, more *ANN* homologous genes were determined in dicots. Obviously, more *LiANN* genes were syntenic with *ANN* genes from *P. trichocarpa* and *S. purpurea*, which was consistent with plant evolutionary relationship.

### Gene structures and conserved motifs of the LiANNs

Further to the clarify the characterizations of the LiANNs, we investigated intron/exon patterns of crape myrtle, Arabidopsis, and rice *ANNs* (Fig. 4). Based on the intron/exon compositions and distributions of the *ANNs*, we classified the ANN members into six groups, namely groups 1–6. The number of *ANN* intron changed from 3 to 6, and the exon ranged from 4 to 6. For example, the *AtANN2/6/7* clustered into group 1 contained 3 introns and 4 exons, while the *LiANN7*, *AtANN1*, and *PtANN4/6/7* consisted of 4 introns and 5 exons. Additionally, the *ANN* members clustered into group 2, except for *PtANN5* and *LiANN6*, comprised only CDSs and no untranslated regions (UTRs). In general, different groups had divergent intron/exon distributions, while the same group shared the relatively similar intron/exon patterns. We also applied MEME online tool to predict ten conserved motifs of the ANN proteins (Supplemental Fig. 5). The number and distribution of conserved motifs were distinctive among the LiANN, PtANN, AtANN, and OsANN members, ranging from 5 to 10. For instance, the LiANN1/3/6 contained 10 conserved motifs, while LiANN5 only had motifs 2, 3, 5, 8, and 9. Although the LiANN2 and 4 were clustered into group 3, LiANN2 shared the similar motif composition with other members and LiANN4 showed little difference with other members. The LiANN1, PtANN4, and AtANN2 clustered into group 4 had the same motif composition and distribution (Fig. 4).

**Fig. 5:**
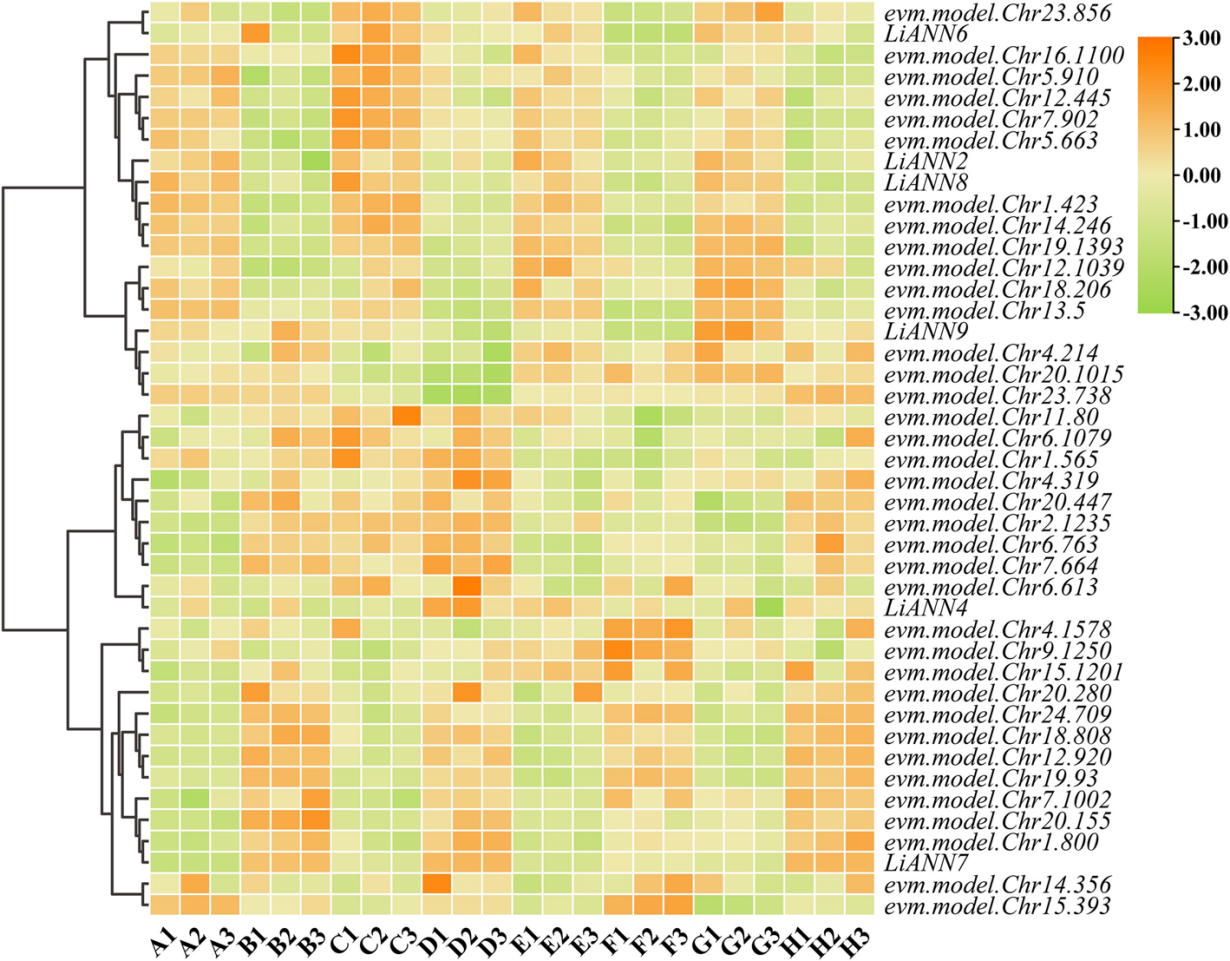
Expression patterns of the *LiANNs* and interaction protein genes during the branching architecture of *L. indica*. Expression data were performed with log2 normalization. The color scale represented relative expression levels. A/B indicated *L. indica* maintaining normal growth; C/D indicated *L. indica* treated by rotation of 180°; E/F indicated *L. indica* treated by rotation of 90°; and G/H indicated *L. indica* treated by rotation of 270°

### Promoter analysis of the *LiANNs*

The gene promoters located upstream of genes can bind to transcription factors, which have a close association with the regulation of gene expression [38]. We identified *cis*-elements for light responsiveness, hormone responsiveness, and stress responsiveness in the 2000 bp upstream regions of the *ANN* promoters (Supplemental Fig. 6). All *ANN* promoters consisted of light responsiveness element, which suggested that ANNs are involved in plant light morphogenesis in plant growth and development. Also, all most of *LiANN* promoters had anaerobic induction element, and *LiANN5* seemed most prominent. In addition, the *LiANN3-5*/*7*/*8* contained GA-responsiveness element, and all *LiANN* promoters were endowed with ABA-responsiveness element. Besides the GA- and ABA-responsiveness elements, the JA- and SA-responsiveness elements were also found in the *LiANN* promoters., Similarly, *LiANN* promoters contained *cis*-elements related to low-temperature, cell cycle regulation, endosperm expression, and meristem specific activation, indicating that LiANNs may respond to diverse environmental stresses and participant in plant growth and development. Moreover, MYB binding sites as the most important IF were detected in the promoters of the *LiANN3*/*6*/*8*/*9* promoters, suggesting that their expression levels are modulated by MYB, and MYB may play a crucial role in transcriptional control of plant growth and stress responses.

**Fig. 6:**
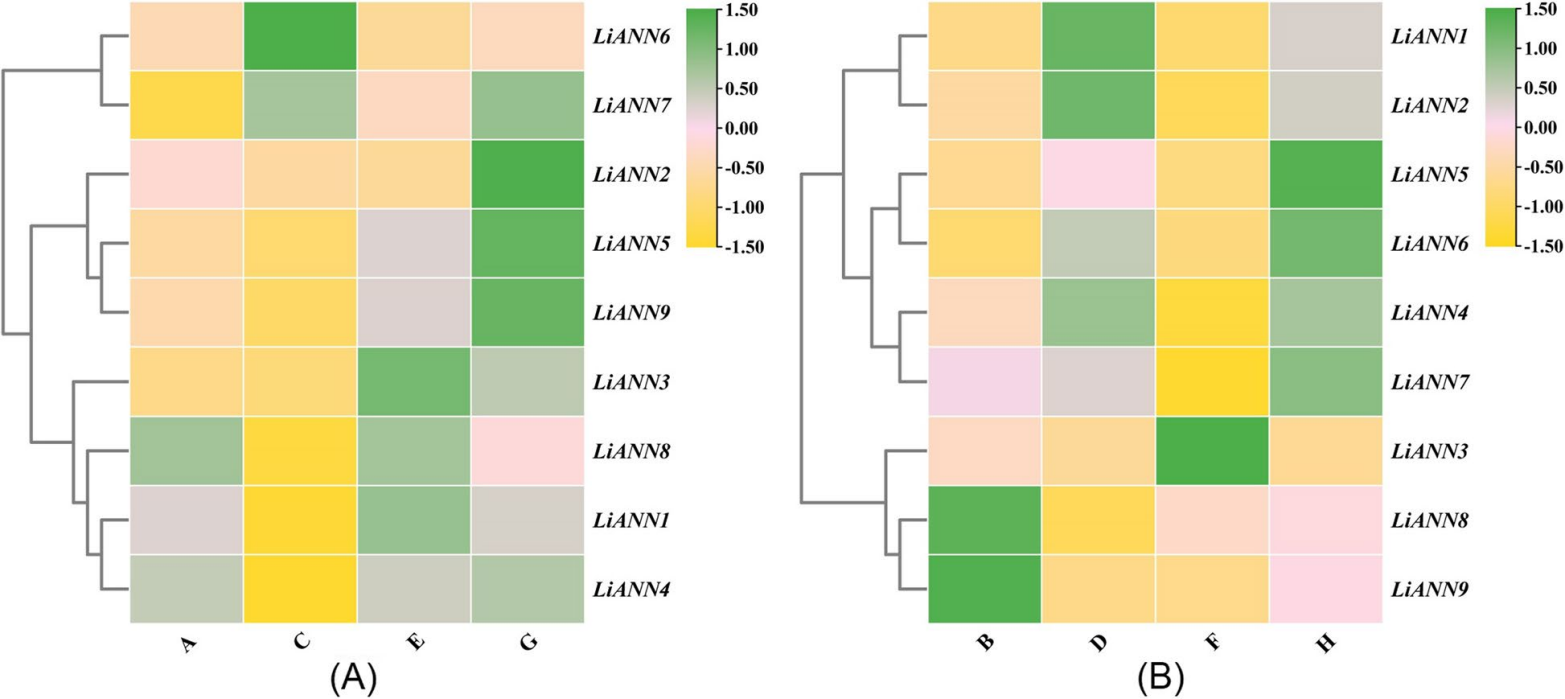
The qRT-PCR analysis of *LiANN* expression profiles at the upper tissues of the bending sites (UTBS) (A) and the lower tissues of the bending sites (LTBS) (B) during the branching architecture of *L. indica*. A/B indicated *L. indica* maintaining normal growth; C/D indicated *L. indica* treated by rotation of 180°; E/F indicated *L. indica* treated by rotation of 90°; and G/H indicated *L. indica* treated by rotation of 270°. Among of them, the A, C, E, and G indicated UTBS, while the B, D, F and H represented LTBS. Expression levels were represented by graded color scale. The green, pink, and dark yellow represented high, middle, and low, respectively. The values of *LiANN* relative expression levels were transformed by log2 and the heatmap was constructed by TBtools software

### Identification of TFs binding *LiANN* promoters

The PlantRegMap online tool and Cytoscape software were used to identify and visualize the putative regulatory network between TFs and *LiANN* genes. The result revealed that *LiANN* genes may be possibly modulated by various kinds of TFs (Supplemental Fig. 7). The evm.model.Chr18.336.1 as characteristic bZIP TFs, homolog with AT5G11260, was identified in the regulation networks, which suggested that *LiANN* expression levels may be simultaneously regulated by bZIP. Also the evm.model.Chr17.1777 considered as homologous gene with AT5G46830 was determined in transcriptional regulation networks of the *LiANNs*, indicating LiANNs may be modulated by bHLHs and are involved in various physiological processes. In addition, the evm.model.Chr1.67 as a homologous gene with AT5G08070, a key enzyme in the biosynthetic pathway of chlorophyll and heme, was speculated to regulate the *LiANNs* expression. Besides bZIP and bHLH, the TCP IFs, cold induced zinc finger protein 2 (C2H2), apetala 2 (AP2), ERF encoding a member of the DREB subfamily A-4 of ERF/AP2 transcription factor family, and squamosa promoter binding protein (SBP) were also identified from the transcriptional regulation networks. These results revealed that LiANNs participant in *L. indica* morphogenesis and physiological processes by complicated regulation of various TFs. The evaluation of putative IFs provides a new perspective for identifying the LiANN functions and profiles as well as discovering the regulatory network.

**Fig. 7:**
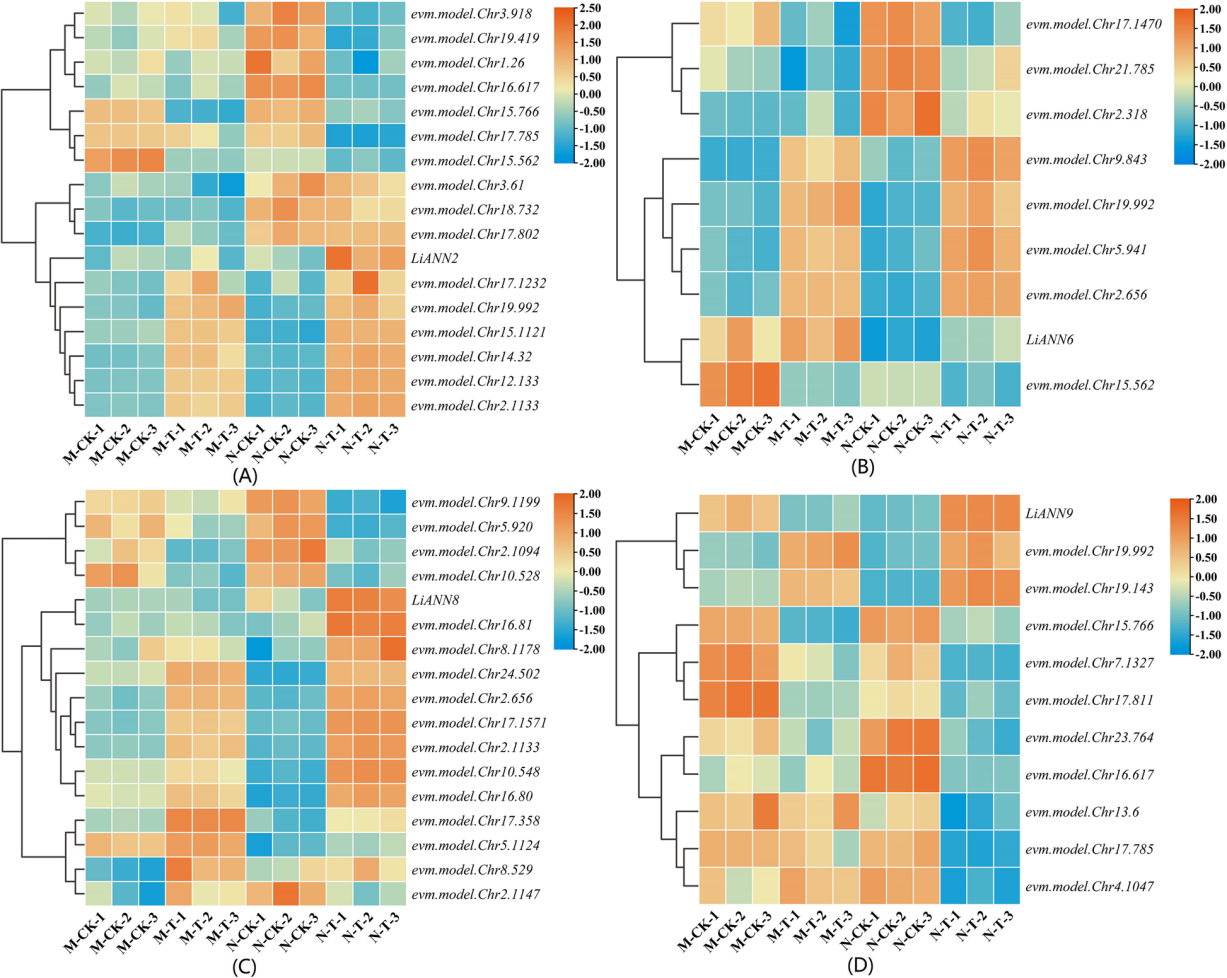
Expression patterns of the differentially expressed *LiANNs*, including *LiANN2* (A), *LiANN6* (B), *LiANN8* (C), and *LiANN9* (D), and corresponding TF genes under salt treatment. Expression data were performed with log2 normalization. The color scale represented relative expression levels. M-CK indicated that salt sensitive variety grown on normal condition; M-T indicated that salt sensitive variety grown on salt condition; N-CK indicated that salt tolerant variety grown on normal condition; and N-T indicated that salt tolerant variety grown under on salt condition

### Protein interaction networks and GO analysis of the LiANNs

Protein interaction network provides an important information on gene functions and patterns. In general, genes with interaction relationship may be involved in the similar signaling transduction or metabolic pathway. As shown in Supplemental Fig. 8, a series of *L. indica* proteins interacting with LiANNs were identified. For example, the evm.model.Chr18.206 as homologous protein with AT2G02090, encoding ETL1SNF2 domain-containing protein/helicase domain-containing protein, was identified to interact with LiANN1. The LiANN2 was predicted to interact with evm.model.Chr6.763 encoding a putative vacuolar H^+^-ATPase. Also, the evm.model.Chr17.1288 encoding exocyst complex component was predicted to interact with LiANN3. The evm.model.Chr21.966.3 encoding a calcineurin-like metallo-phosphoesterase superfamily protein and the evm.model.Chr1.554 encoding a polypeptide with K-homology (KH) RNA-binding modules were found to interact with LiANN7 and LiANN9, respectively. In addition, a series of functional proteins were identified from the interaction network. For example, the evm.model.Chr20.447 encoded a transcription factor IIb, the evm.model.Chr12.920 encoded an alpha/beta hydrolase, and the evm.model.Chr23.738 encoded a SWEET sucrose efflux transporter family protein.

**Fig. 8:**
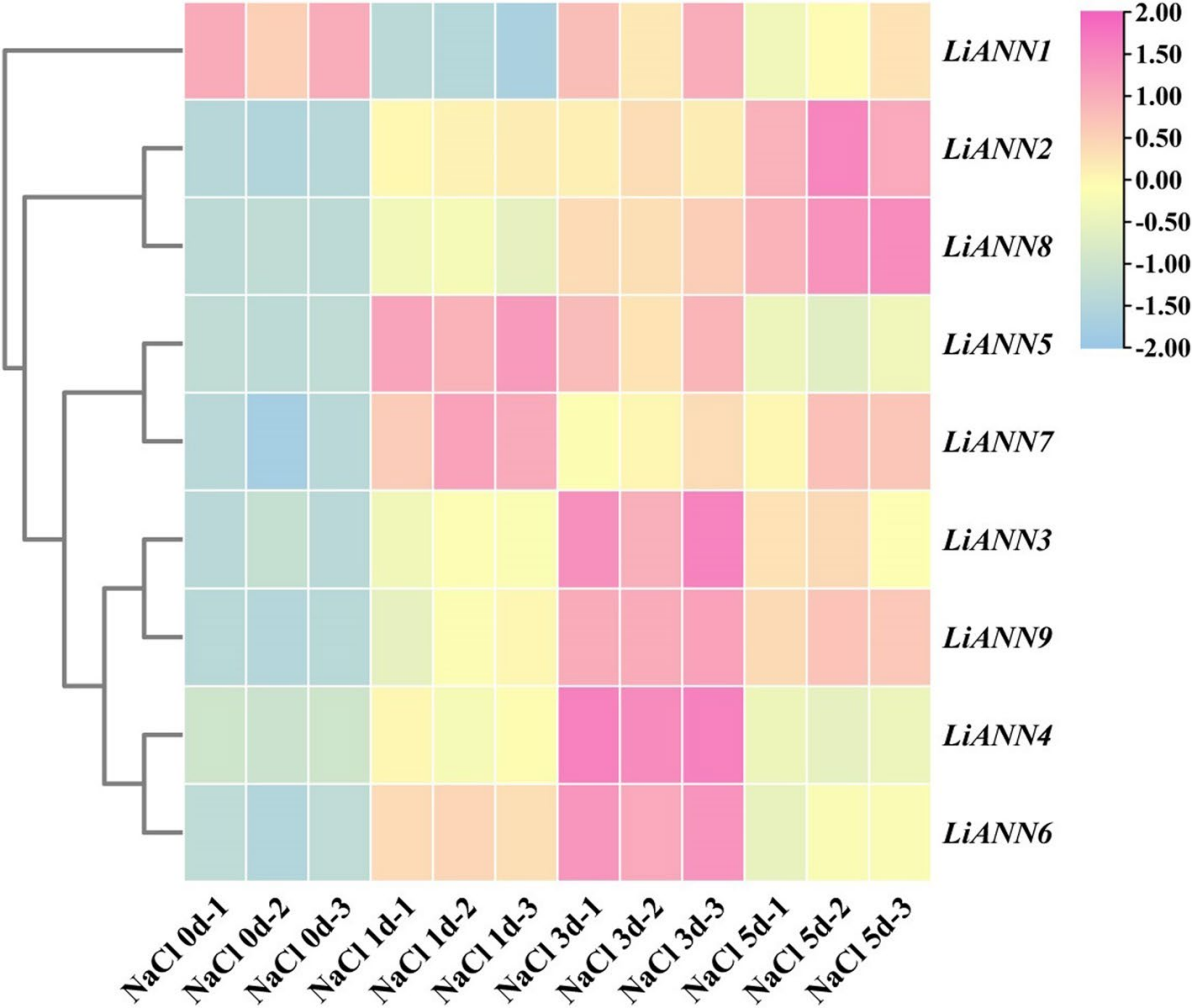
Expression profiles of *LiANN* genes under NaCl stress. The qRT-PCR was used to illustrate the transcript levels of *LiANN* genes under NaCl stress with three biological and three technical replicates. Expression levels were represented by graded color scale. The rose hermosa, yellow, and light blue represent high, middle, and low, respectively. The values of *LiANN* relative expression levels were transformed by log2 and the heatmap was constructed by TBtools software

GO provided insight into investigation of gene function, and *LiANNs* were effectively annotated and identified GO terms using GO annotation file and TBtools software. GO analysis revealed that *LiANNs* were significantly enriched in molecular functions, biological processes, and cellular components (Supplemental Fig. 9). In the molecular function category, the *LiANNs* were enriched in phospholipid binding (GO:0008289), calcium-dependent phospholipid binding (GO:0005543), lipid binding (GO:0008289), calcium ion binding (GO:0005509), metal ion binding (GO:0046872), cation binding (GO:0043169), and metal ion binding (GO:0046872). In the biological process category, the *LiANNs* were enriched in exocyst (GO:0000145), cell cortex (GO:0005938), vesicle tethering complex (GO:0099023), cell periphery (GO:0071944), and cytoplasm (GO:0033655). In the cellular component category, the *LiANNs* were enriched in vesicle-mediated transport (GO:0016192), establishment of localization (GO:0051649), organonitrogen compound biosynthetic process (GO:1,901,566), and cellular process (GO:0009987). In conclusion, the GO enrichment analysis indicated that LiANNs play an essential role in formation of ion channels, ion transports, phosphorylation, and phospholipid binding. These results will provide a new direction for future research on characterizations and functions of the LiANNs.

### RNA-seq analysis of the *LiANNs* on branching architecture

To explore the putative roles of the LiANNs on branching architecture of *L. indica*, we analyzed the differentially expressed genes (DEGs) of the *LiANNs* in the transcriptomic database. We found that the *LiANN2*/*4*/*6*–*9* expression levels are significantly changed in branching architecture and they may be potential genes associated with branching architecture. To investigate the putative mechanism of branching architecture, the clustering heat map of the *LiANN2*/*4*/*6*–*9* and interaction protein genes were constructed (Fig. 5). A total of 6 *LiANNs* and 37 interaction protein genes were determined as the DEGs during the branching architecture of *L. indica*. The *evm.model.Chr19.1393* (At annotation CYCD2) and *evm.model.Chr14.246* (At annotation CYCA3) displayed similar expression profiles with *LiANN2/8*, possessing higher expression levels in stages A/C/E/G and lower expression levels in stages B/D/F/H. Also, the expression of *evm.model.Chr23.856* (At annotation PANS2) accumulated in stages C/E/G, sharing the similarity with *LiANN6*. The *evm.model.Chr4.214* (At annotation phosphate transporter 2), *evm.model.Chr20.1015* (At annotation GLX1), and *evm.model.Chr23.738* (At annotation SWEET11) shared the similar expression levels with *LiANN9*, having the highest expression levels in stages G/H. In addition, the *evm.model.Chr7.1002* (At annotation aldehyde dehydrogenase 6B2), *evm.model.Chr20.155* (At annotation EXO70A2), and *evm.model.Chr1.800* (At annotation SNF7) presented the same mRNA levels with *LiANN7*, with the higher expression in stages B/D/F/H.

To further understand the relationship between *LiANN2*/*4*/*6–9* and corresponding TF genes during the branching architecture, we simultaneously evaluated the expression profiles of these genes. For *LiANN2*, a total of 29 putative TF gene expression levels were significantly changed. The *evm.model.Chr6.1557* (ortholog of Dof protein, AT3G50410), *evm.model.Chr16.617* (ortholog of TCP24, AT1G30210), and *evm.model.Chr1.26* (ortholog of Dof protein, AT5G62940) were higher expressed in stages A/C/E/G, possessing same expression changes with *LiANN2*, whereas the *evm.model.Chr18.732* (ortholog of shoot gravitropism 5, AT2G01940), *evm.model.Chr17.802* (ortholog of NAC043, AT2G46770), and *evm.model.Chr19.419* (ortholog of NAC043, AT4G28500) showed the opposite expression levels with *LiANN2*, having the higher expression in stages B/D/H (Supplemental Fig. 10A). For *LiANN4*, 83 putative TF gene expression levels were dominantly detected in the branching architecture of all developmental stages, including *evm.model.Chr19.943* (ortholog of ERF109, AT4G34410), *evm.model.Chr17.1648* (ortholog of ERF105, AT5G51190), *evm.model.Chr18.490* (ortholog of WIND1, AT1G78080), and *evm.model.Chr6.1422* (ortholog of ERF/AP2, AT1G12630) (Supplemental Fig. 10B). For *LiANN6*, a total of 27 putative TF gene expression levels were significantly changed. For example, the *evm.model.Chr6.636* (RTV1, AT1G49480 orthologue) and *evm.model.Chr4.522* shared the similar transcript profiles with *LiANN6*, with the higher expression in stages C/E/G*.* However, *evm.model.Chr5.941* (bHLH, AT4G14410 orthologue) and *evm.model.Chr15.562* (DOF1, AT1G51700 orthologue) were determined to have lower mRNA levels in stages C/E/G (Supplemental Fig. 10C). For *LiANN7*, 41 putative TFs were identified to be involved in branching architecture of *L. indica* (Supplemental Fig. 10D). The cluster heatmap of TF gene expression patterns were divided into two clustered, one of which contained 19 TF genes; such as *evm.model.Chr17.1648* orthologous with ERF104 (AT5G61600), *evm.model.Chr2.1133* orthologous with ERF/AP2 (AT2G40340), and *evm.model.Chr24.824* orthologous with ENY (AT5G66730); the other contained 23 TF genes, such as *evm.model.Chr18.732* orthologous with SGR5 (AT2G01940) and *evm.model.Chr10.709* orthologous with ESE3 (AT5G25190). Clustering network analysis of *LiANN8* and corresponding IF gene expression patterns showed that they gathered into two distinct groups (Supplemental Fig. 10E). The *LiANN8*, *evm.model.Chr8.1178* (GATA4, AT3G60530 orthologue) and *evm.model.Chr3.669* (SOL2, AT4G14770 orthologue), were clustered together in the dendrogram. The *LiANN9* expression pattern had similarity with *evm.model.Chr11.1055* (GBF1, AT4G36730 orthologue), *evm.model.Chr6.636*, and *evm.model.Chr15.766* (DOF4, AT4G21030 orthologue) (Supplemental Fig. 10F), which implicated that these IFs have a positive regulation on *LiANN8* expression.

### qRT-PCR analysis of the *LiANNs* on branching architecture

To illustrate putative roles of the LiANNs, the qRT-PCR was used to analyze expression levels of the *LiANNs* during branching architecture of *L. indica*. The *LiANN* expression levels at UTBS untreated by rotation was used as calibrator to determine their relative expression on branching architecture. For UTBS expression profiles, treatment by rotation of 180°showed significant down-regulation in the mRNA levels of *LiANN1*, *4*, and* 8*, while *LiANN2*, *3*,* 5*, and *9* represented no significant changes in expression levels, and *LiANN6* and *7* expression showed dominant up-regulation (Fig. 6A). In addition, the *LiANN 3*,* 5*, *7*, and *9* showed a significant increase in transcript levels after treatment by rotation of 90°, while other *LiANN* gene expression levels had no significant changes (Fig. 6A). Moreover, the treatment with rotation of 270°induced strong improvement in transcript levels of *LiANN 2*, *3*,* 5*, *7*, and *9*, while *LiANN 1*, *4*,* 6*, and *8*, showed comparatively unremarkable changes in transcript levels (Fig. 8A). Similarity with UTBS expression, the LTBS expression was observed with little/marginal change in transcript level. Except for *LiANN 3* and *7* with no significant changes and *LiANN 8* and *9* with marginal increases in mRNA levels, the *LiANN1*, *2*, and *4–6* showed nearly 2–threefold increase in the level of transcripts after treatment by rotation of 180° (Fig. 6B). In addition, the down-regulation in *LiANN2*, *4*, and *7–9* was to a lesser level with down to 2–fourfold decrease in the transcript levels after treatment by rotation of 90° (Fig. 6B). For treatment by rotation of 270°, the trends in *LiANN8* and *9* were a significant decrease in transcript levels, while *LiANN1*, *2*, and *4–7* showed an increase in expression levels (Fig. 6B). In summary, the results indicated that the *LiANN* expression levels was dominant change, and they could play a vital role in the process of crape myrtle branching architecture.

### RNA-seq analysis of the *LiANNs* on salt treatment

To further validate whether the *LiANN* gene expression was affected by salt treatment, the *L. indica* samples treated by salt treatment were performed for RNA-seq. When the *L. indica* was treated with salt, the expression of *LiANN2*/*6*/*8*/*9* was significantly changed. The expression patterns of the *LiANN2* and corresponding TF genes were clustered into four groups (Fig. 7A). Genes clustered into group 1 containing *evm.model.Chr3.918* (SND4, AT4G29230 orthologue), *evm.model.Chr19.419* (NAC073, AT4G28500 orthologue), *evm.model.Chr1.26* (HCA2, AT5G62940 orthologue), and *evm.model.Chr16.617* (TCP24, AT1G30210 orthologue) were significantly down-regulated in salt tolerant variety of *L. indica* after salt treatment. Also, the expression levels of *evm.model.Chr15.766*, *evm.model.Chr17.785*, and *evm.model.Chr15.562* (ADOF1, AT1G51700 orthologue) classified into group 2 were significantly decreased in both salt sensitive and salt tolerant varieties when *L. indica* was treated by salt treatment. In addition, the transcript levels of *evm.model.Chr3.61* (CRF4, AT4G27950 orthologue), *evm.model.Chr18.732* (IDD15, AT2G01940 orthologue), and *evm.model.Chr17.802* (NAC043, AT2G46770 orthologue) were significantly accumulated in salt tolerant variety before and after salt treatment. After salt treatment, transcript of *LiANN2* in salt sensitive variety had no significant difference, while it was significantly promoted in salt tolerant variety. The expression abundances of *evm.model.Chr17.1232* (NAC030, AT1G71930 orthologue), *evm.model.Chr19.992* (TFIIIA, AT1G72050 orthologue), *evm.model.Chr15.1121*, *evm.model.Chr14.32* (ERF9, AT5G44210 orthologue), *evm.model.Chr12.133* (CDF5, AT1G69570 orthologue), and *evm.model.Chr2.1133* were dominantly improved in both salt sensitive and salt tolerant varieties after salt treatment. The result revealed that LiANN2 can be considered as marker gene in response to salt stress.

In response to salt treatment, *LiANN6* showed similar expression pattern with *evm.model.Chr15.562*, with the higher expression in salt sensitive variety and lower expression in salt tolerant variety. The *evm.model.Chr9.843* (ABF2, AT1G45249 orthologue), *evm.model.Chr19.992*, *evm.model.Chr5.941*, and *evm.model.Chr2.656* were clustered together, with higher transcript accumulations in both salt sensitive and salt tolerant varieties under salt treatment (Fig. 7B). For *LiANN8* and related TF gene expression profiles, the cluster dendrogram was classified into three groups. The *LiANN8* and *evm.model.Chr16.81* (NAC025, AT1G61110 orthologue) were endowed with similar expression pattern, with higher accumulation in salt tolerant variety after salt treatment, which implicated that evm.model.Chr16.81 may positively regulate the *LiANN8* expression in response to salt stress. Interestingly, compared with *LiANN8*, the *evm.model.Chr9.1199* (MYBCDC5, AT1G09770 orthologue), *evm.model.Chr5.920*, *evm.model.Chr2.1094* (MYB, AT3G11280 orthologue), and *evm.model.Chr10.528* (ERF014, AT1G44830 orthologue) possessed the opposite expression patterns, which suggested that they can be considered as the negative TFs that modulate the *LiANN8* expression under the salt stress (Fig. 7C). The *LiANN9*, *evm.model.Chr19.992*, and *evm.model.Chr19.143* (NAC062, AT3G49530 orthologue) were clustered into same group, with higher expression levels in salt tolerant variety after salt treatment (Fig. 7D). In addition, the expression level of *LiANN9* was down-regulated in salt sensitive variety after salt treatment, whereas *evm.model.Chr19.992* and *evm.model.Chr19.143* expression was up-regulated after salt treatment. The expression levels of *evm.model.Chr15.766*, *evm.model.Chr7.1327* (NAC087, AT5G18270 orthologue), and *evm.model.Chr17.811* (NAC004, AT1G02230 orthologue), possessing oppositive expression profile with *LiANN9*, were dominantly down-regulated after salt stress, suggesting that these TFs may negatively regulate *LiANN9* expression in response to salt treatment (Fig. 7D).

### qRT-PCR analysis of the *LiANNs* under the NaCl stress

Gene expression profiles of *LiANNs* under NaCl stressby qRT-PCR analysis were investigated for *LiANNs* in crape myrtle. The relative expression levels were presented with clusters using log2 fold-change values. The 8 of 9 *LiANN* genes, including *LiANN2-9* were up-regulated under simulated NaCl treatment. Among of them, *LiANN3*, *4*, *6*, and *9* showed highly specific expression levels after NaCl treatment for 3 d; *LiANN5* and* 7* represented the highest transcript levels after NaCl treatment for 1 d; and *LiANN2* and* 8* had significantly highest mRNA expression levels after NaCl treatment for 5 d (Fig. 8).

## Discussion

The ANNs are found in the plant kingdom, which play crucial roles in regulating plant growth and development and are involved in various abiotic stresses [5]. Previous studies were constrained on only Arabidopsis, tomato, rice, and maize ANNs [18], and little is systematically reported on the characterization and function of the ANN family gene in crape myrtle. In this study, a genome-wide identification with strict standards revealed 9 *ANN* genes from *L. indica* and named *LiANN1-9* based on the chromosome localization. Previous studies showed that the plant ANN family members were small, and the complexity as well as diversity of ANNs was low [39]. Here, a total of 39 ANN members were divided into 3 subfamilies (Fig. 1), which was consistent with a previous report on poplar [4]. However, Arabidopsis, tomato, and rice ANN proteins were classified into five groups [39], and wheat and Brassicaceae ANNs were divided into six groups [3, 40]. In fact, based on the principle of Arabidopsis ANN family grouping, the AtANNs were divided into five groups: AtANN3 group, AtANN4 group, AtANN5 group, AtANN8 group and AtANN1/2/6/7 group. For example, the AtANN3 and LiANN2/4, or AtANN8 and LiANN3/5/6 were clustered into same branch (Fig. 1), which suggested that LiANN family member can also be divided into five groups according to the principle of Arabidopsis ANN family grouping. Various ANN family members contained a series of conserved domains, and all ANN proteins retained a N- or C-terminal protein kinase domains. In this study, we found LiANN2-7 are composed of conserved GWGT domain, while the GWGT domain of the LiANN1/8/9 was not absolutely conservative, which implied the LiANN2-7 possess relatively stronger binding activity with Ca^2+^ when compared with LiANN1/8/9 (Supplemental Fig. 2). In addition, the LiANN2/4/6/7 were identified to possess dominant the GTP binding domain (GXXG), a typical structure of GTPase, whereas other LiANNs had no conservative GXXG domain (Supplemental Fig. 2), suggesting LiANN2/4/6/7 play a vital function in ANN nucleotide-binding and hydrolyzation [41]. Previous reports showed that the maize ANNs have the similar function of hydrolyzing ATP and GTP, but their ability to GTP affinity was remarkably lower than cotton ANN1. Also, the cotton ANN2 and maize ANN33/35 were endowed with characterization of GTP-binding motif overlapping with the Ca^2+^-binding site, implicating a special regulatory relationship occurs in Ca^2+^ and GTPase activities [42]. Similarity, the LiANN members had different motifs (Fig. 4), implying there is functional diversification among LiANN members. However, the exact functions of the LiANN members in ATPase and GTPase activities, Ca^2+^ binding ability, and corresponding profiles need to be investigated in the further study.

Gene duplication events commonly reveal generation and function of family gene during the evolutionary process [43, 44]. The syntenic relationship revealed that segmental duplication (2 *LiANN* gene pairs) and tandem duplication (1 *LiANN* gene pair) events were seem to have contributed to the expansion of the *LiANN* genes (Fig. 2). In general, gene duplication events were speculated to be essential for the adaptability of plant to complex environmental conditions [45]. We can speculated that the LiANNs with collinearity relationship play an important role in response to various environment during the evolutionary process of crape myrtle. The tandem and segmental duplicate gene pairs in *LiANNs* underwent purifying selection based on the calculator of Ka/Ks. It was also identified that most of the *LiANN* genes are disrupted by 4–6 introns (Fig. 4). This was consistent with the Arabidopsis, rice, and poplar *ANN* gene structure patterns, suggesting that most *ANN* genes in land plants contained 4–6 introns. Interestingly, some *LiANNs* shared with similar gene structures had collinearity relationship (Fig. 2 and Fig. 4), which was consistent with previous reports that syntenic gene pairs experiencing purifying selection may generate genes with conserved characterizations and functions [46]. In addition, exon/intron compositions and distributions of the *LiANNs* had a strong correlation with phylogenetic tree, and the LiANNs clustered into the same branch shared the similar gene structure (Fig. 4), which was consistent with previous studies [39]. Totals of 0, 0, 3, 9, 9, and 11 orthologous *ANN* gene pairs were found through collinearity analysis of crape myrtle versus rice, grape, Arabidopsis, eucalyptus, poplar, and willow (Fig. 3), suggesting that large-scale expansion generated before the divergence of these four comparisons. In addition, the evolutionary relationship among crape myrtle, eucalyptus, poplar, and willow may be the closest among these species. The collinearity analysis of crape myrtle, eucalyptus, poplar, and willow showed that *LiANN7-9* are identified as genes with various syntenic gene pairs, suggesting these genes may play a key role in the ANN family gene expansion and evolution.

Accumulating evidence revealed that gene expression was generally correlated with divergences in promoter regions [46, 47]. ABA as an essential plant hormone participants in plant growth regulation and stress resistance. Therefore, exogenous application of ABA can modulate the influences of various environmental conditions [49]. Here, all *LiANN* promoters had ABA-responsiveness (Supplemental Fig. 6), and 9 *LiANN* genes might be induced by exogenous ABA, which was consistent with other plant *ANN* genes [3]. For example, the expression level of *OsANN3* was stimulated by ABA and drought stresses, and overexpression of *OsANN3* improved drought tolerance through increasing ABA accumulation and promoting stomata closure [12]. SA as a key hormone in plant immunity participants in internal defense signal transduction of hypersensitive responses and cell death [50]. In this study, *LiANN6*/*7*/*9* promoters contained SA-responsive *cis*-elements and these genes could be affected by exogenous SA (Supplemental Fig. 6). Compared with *P. trichocarpa* and *O. sativa*, the number of SA-responsive *cis*-elements in *L. indica* were significant less (Supplemental Fig. 6), which indicated that functional diversities may occur in *L. indica*, *P. trichocarpa* and *O. sativa* ANN. Up to now, previous studies showed that pepper *ANN9* (*CaANN9*) induced by *Bemisia tabaci* and MeJA, and CaANN9 play an important role in plant tolerance to *B. tabaci* by JA signal transduction [51]. *Brassica rapa* ANN family gene was also involved in JA signal transduction [51], and *A. thaliana* AtANN1/2 responded to *Meloidognye incognita* and manipulated host immune response by interacting with MiMIF-2, a novel effector [52]. Here, all *LiANN* promoters endowed with JA-responsive *cis*-elements indicated these genes may be affected by exogenous JA (Supplemental Fig. 6). The number of JA-responsive *cis*-elements in *L. indica* was significant more than that in Arabidopsis, rice, and poplar (Supplemental Fig. 6), which indicated that JA signal transduction in *L. indica* play an essential role in response to abiotic stress. GA, a group of tetracyclic diterpenoids, perform function in regulating several aspects of growth and development of higher plants [53]. Here, the promoters of *LiANN3*-*5*/*7*/*8* had GA-responsive elements (Supplemental Fig. 6), which indicated that these genes tend to integrate *L. indica* growth and development by activating GA signal pathway.

The developing molecular evidences have indicated that plant ANNs are widely involved in growth and development, with each ANN having a unique function [54]. For example, the cotton GhAnn2 was closely associated with fiber differentiation and development [20]. The AtANN5 significantly participated in transition from the vegetative to reproductive phase and embryogenesis [55]. The *Medicago truncatula* ANN1 (MtANN1) was proposed to be involved in the early of cortical cell cycle activation [56]. The *Triticum timopheevi *TtANND1 regulated by serine carboxypeptidase-like protein 5 (TtGS5) determined grain size and weight [57]. The branching is a major developmental process that is crucial to plant reproduction and adaptation to prevailing environmental conditions [58]. The *L. indica* as a kind of important ornamental plants possessed dominant branching characteristics, while little study on regulatory mechanism of branching architecture in *L. indica.* Here, the *LiANN2*/*4*/*6–9* considered as the DEGs and a series of interaction proteins were identified from the RNA-seq (Fig. 5). The *LiANN4* expression profiles showed similarity with *evm.model.Chr6.613* (At annotation AT3G18820) possessing dominant GTPase activity. The *LiANN6* had the similar expression patterns with *evm.model.Chr23.856* homologous protein with AT5G12360 encoding a protein that protects meiotic centromere cohesion. The *LiANN7* displayed similar expression pattern with *evm.model.Chr1.800* (At annotation AT2G06530) belonging to SNF7 family protein. which indicated that these genes are associated with branching architecture of crape myrtle. Taken together, these DEGs and corresponding interaction proteins were likely to play important roles in branching architecture of crape myrtle. In addition, to further identify the putative regulatory mechanism of branching architecture in crape myrtle, the expression patterns of differentially expressed *LiANNs* and corresponding TFs were investigated (Supplemental Fig. 10). Comparing the RNA-seq data, a series of TFs associated with branching architecture were identified and mapped to the Arabidopsis genome. For example, *evm.model.Chr19.943* (At annotation AT4G34410) and evm.model.Chr17.1648 (At annotation AT5G51190), encoding ERF family proteins, had the similar expression patterns with *LiANN4*. The *evm.model.Chr19.1539*, counterpart for AT5G46590 encoding NAC096, were investigated to possess the similar expression patterns with *LiANN9*. Moreover, the qRT-PCR results showed that the *LiANN* expression levels was dominant change and they could play a vital role in the process of crape myrtle branching architecture (Fig. 6). All above results significantly implied that these IFs and *LiANNs* as putative candidates have a close relationship with crape myrtle branding architecture. The branching architecture of crape myrtle provided a new direction for exploring the regulatory mechanism of branding architecture in crape myrtle.

The plant *ANN* expression levels are closely associated with stress treatment [50]. Arabidopsis *AtANN1/4* expression were affected by salt stress and considered as a positive regulator of salinity tolerance [24, 59]. *B. juncea BjANN2* was characterized for its function in salt tolerance, and *B. rapa Bra034404* expression exhibited a high accumulation under salt treatment [26, 60]. Here, RNA-seq data showed that *LiANN2*/*6*/*8*/*9* were significantly induced under the salt treatment (Fig. 7). Among of them, the transcript levels of *LiANN2*/*8* in salt tolerant variety were significantly improved, while them in salt sensitive variety showed no remarkable change, suggesting *LiANN2*/*8* as putative marker gene plays a crucial role in response to salt stress. Also, *LiANN6* was lowly expressed in salt tolerant variety before and after salt stress, and *LiANN9* was up-regulated in salt tolerant variety and down-regulated in salt sensitive variety under salt treatment, implying they have potential role of salt-stimulative expression. To further understand the regulatory modules of *LiANNs* under salt treatment, we identified large numbers of candidate TF genes whose expression patterns corresponding to *LiANNs* in *L. indica* (Fig. 7). Among these candidates, the *evm.model.Chr17.1232*, *evm.model.Chr19.992*, *evm.model.Chr15.1121*, *evm.model.Chr14.32*, *evm.model.Chr12.133*, and *evm.model.Chr2.1133* had similar expression patterns with *LiANN2* in salt tolerant variety under salt treatment. Also, the *evm.model.Chr16.81* shared the similar expression profile with *LiANN8*, while the *evm.model.Chr9.1199*, *evm.model.Chr5,920*, *evm.model.Chr2.1094*, and *evm.model.Chr10.528* endowed with oppositive expression profiles when they were compared with *LiANN8*. We speculated that the evm.model.Chr16.81 may positively regulate *LiANN8* expression, and evm.model.Chr9.1199, evm.model.Chr5.920, evm.model.Chr2.1094, and evm.model.Chr10.528 negatively regulate *LiANN8* expression. Additionally, similar expression patterns were identified among *LiANN9*, *evm.model.Chr19.992*, and *evm.model.Chr19.143* when salt tolerant variety was treated by salt treatment, which indicated that evm.model.Chr19.992 and evm.model.Chr19.143 play a positive role in regulation of *LiANN9* expression. Additionally, the qRT-PCR results also documented that the some* LiANN* mRNA levels were induced by salt treatment, which further suggested that some LiANNs are involved in response to salt treatment (Fig. 8). These suggested that LiANN members may be modulated by different regulators and adapted different regulation networks to respond to salt stress.

## Conclusions

In this study, we identified 9 *ANN* genes from *L. indica* and characterized their conserved Ca^2+^ and GTPase binding domains. The LiANN members were divided into 2 subfamilies, and each branch possesses specific intron/exon distribution and motif composition. The *LiANN* genes are unevenly distributed on the 7 chromosomes of *L. indica*. It was worth noting that 1 tandem duplication event and 2 segmental duplication events were identified from the *LiANNs*, which suggested that tandem and segmental duplication events are the important driving force for *LiANN* evolution. In addition, we investigated the syntenic relationship of *ANN* genes between *L. indica* and six representative species. Furthermore, we identified gene expression patterns during the branching architecture and salt treatment. According to the RNA seq and qRT-PCR analysis, some *LiANNs* and corresponding TFs have been identified to be associate with branching architecture and salt tolerance in *L. indica*. This study lays a foundation for further studies of the *LiANN* genes.

### Supplementary Information

Below is the link to the electronic supplementary material.

Supplementary file1 (DOCX 4232 kb)

## Data Availability

The sequenced raw reads generated during the current study have been submitted to the China National GeneBank DataBase (CNGBdb) (https://db.cngb.org/) with the accession number CNP0003990 and CNP0003991.

## References

[1] Jami SK, Clark GB, Ayele BT (2012). Genome-wide comparative analysis of annexin superfamily in plants. PLoS ONE.

[2] Clark GB, Sessions A, Eastburn DJ (2001). Differential expression of members of the annexin multigene family in Arabidopsis. Plant Physiol.

[3] Xu L, Tang Y, Gao S (2016). Comprehensive analyses of the annexin gene family in wheat. BMC Genomics.

[4] Wei H, Movahedi A, Liu G (2022). Genome-wide characterization and abiotic stresses expression analysis of annexin family genes in poplar. Int J Mol Sci.

[5] Yadav D, Boyidi P, Ahmed I (2018). Plant annexins and their involvement in stress responses. Environ Exp Bot.

[6] Clark GB, Morgan RO, Fernandez MP (2012). Evolutionary adaptation of plant annexins has diversified their molecular structures, interactions and functional roles. New Phytol.

[7] Saad RB, Ben Romdhane W, Ben Hsouna A (2020). Insights into plant annexins function in abiotic and biotic stress tolerance. Plant Signal Behav.

[8] Shen F, Ying J, Xu L, et al. Characterization of Annexin gene family and functional analysis of RsANN1a involved in heat tolerance in radish (*Raphanus sativus* L.). Physiol Mol Biol Pla. 2021; 27:2027–2041. 10.1007/s12298-021-01056-5 PMC8484430 34629776

[9] Gerke V, Creutz CE, Moss SE (2005). Annexins: linking Ca^2+^ signalling to membrane dynamics. Nat Rev Mol Cell Bio.

[10] Laohavisit A, Shang Z, Rubio L (2012). Arabidopsis annexin1 mediates the radical-activated plasma membrane Ca^2+^- and K^+^-permeable conductance in root cells. Plant Cell.

[11] Hu NJ, Yusof AM, Winter A (2008). The crystal structure of calcium-bound an-nexinGh1 from *Gossypium hirsutum* and its implications for membrane binding mechanisms of plant annexins. J Biol Chem.

[12] Li X, Zhang Q, Yang X (2019). OsANN3, a calcium-dependent lipid binding annexin is a positive regulator of ABA-dependent stress tolerance in rice. Plant Sci.

[13] Mu C, Zhou L, Shan L, et al. Phosphatase GhDsPTP3a interacts with annexin protein GhANN8b to reversely regulate salt tolerance in cotton (*Gossypium spp*.). New Phytol. 2019;223:1856–1872. 10.1111/nph.15850 30985940

[14] Gorecka KM, Konopka-Postupolska D, Hennig J (2005). Peroxidase activity of annexin 1 from *Arabidopsis thaliana*. Biochem Bioph Res Co.

[15] Konopka-Postupolska D, Clark G, Goch G (2009). The role of annexin 1 in drought stress in Arabidopsis. Plant Physiol.

[16] Deng Z, Chen J, Leclercq J (2016). Expression profiles, characterization and function of HbTCTP in rubber tree (*Hevea brasiliensis*). Front Plant Sci.

[17] Qiao B, Zhang Q, Liu D (2015). A calcium-binding protein, rice annexin OsANN1, enhances heat stress tolerance by modulating the production of H_2_O_2_. J Exp Bot.

[18] Zhang F, Jin X, Wang L (2016). A cotton annexin affects fiber elongation and secondary cell wall biosynthesis associated with Ca^2+^ influx, ROS homeostasis, and actin filament reorganization. Plant Physiol.

[19] Li B, Li DD, Zhang J (2013). Cotton AnnGh3 encoding an annexin protein is preferentially expressed in fibers and promotes initiation and elongation of leaf trichomes in transgenic Arabidopsis. J Integr Plant Biol.

[20] Tang W, He Y, Tu L (2014). Down-regulating annexin gene *GhAnn2* inhibits cotton fiber elongation and decreases Ca^2+^ influx at the cell apex. Plant Mol Biol.

[21] Zhu J, Yuan S, Wei G (2014). Annexin5 is essential for pollen development in Arabidopsis. Mol Plant.

[22] He M, Yang X, Cui S, et al. Molecular cloning and characterization of annexin genes in peanut (*Arachis hypogaea* L.). Gene 2015;568:40–49. 10.1016/j.gene.2015.05.004 25958350

[23] Feng YM, Wei XK, Liao WX (2013). Molecular analysis of the annexin gene family in soybean. Biol Plantarum.

[24] Huh SM, Noh EK, Kim HG (2010). Park, Arabidopsis annexins AnnAt1 and AnnAt4 interact with each other and regulate drought and salt stress responses. Plant Cell Physiol.

[25] Wang X, Ma X, Wang H (2015). Proteomic study of microsomal proteins reveals a key role for Arabidopsis annexin 1 in mediating heat stress-induced increase in intracellular calcium levels. Mol Cell Proteomics.

[26] Ahmed I, Yadav D, Shukla P (2017). Constitutive expression of *Brassica juncea* annexin, AnnBj2 confers salt tolerance and glucose and ABA insensitivity in mustard transgenic plants. Plant Sci.

[27] He F, Gao C, Guo G (2019). Maize annexin genes *ZmANN33* and *ZmANN35* encode proteins that function in cell membrane recovery during seed germination. J Exp Bot.

[28] Li S, Zheng T, Zhuo X (2020). Transcriptome profiles reveal that gibberellin-related genes regulate weeping traits in crape myrtle. Hortic Res.

[29] Li S, Zheng T, Zhuo X (2020). Isolation of the crape myrtle decreased apical dominance gene *LfiDAD2* and characterization of its function in the control of axillary branching. Sci Hortic.

[30] Ijaz R, Ejaz J, Gao S (2017). Overexpression of annexin gene *AnnSp2*, enhances drought and salt tolerance through modulation of ABA synthesis and scavenging ROS in tomato. Sci Rep.

[31] Letunic I, Bork P (2007). Interactive Tree of Life (iTOL): an online tool for phylogenetic tree display and annotation. Bioinformatics.

[32] Fan L, Xu L, Wang Y, et al. Genome- and Transcriptome-wide characterization of bZIP gene family identifies potential members involved in abiotic stress response and anthocyanin biosynthesis in radish (*Raphanus sativus* L.). Int J Mol Sci. 2019;20:6334. 10.3390/ijms20246334 PMC6941039 31888167

[33] Chen C, Chen H, Zhang Y (2020). TBtools: an integrative toolkit developed for interactive analyses of big biological data. Mol Plant.

[34] Lescot M, Déhais P, Thijs G (2002). PlantCARE, a database of plant cis-acting regulatory elements and a portal to tools for in silico analysis of promoter sequences. Nucleic Acids Res.

[35] Shannon P, Markiel A, Ozier O (2003). Cytoscape: a software environment for integrated models of biomolecular interaction networks. Genome Res.

[36] Guo Z, Ma D, Li J (2022). Genome-wide identification and characterization of aquaporins in mangrove plant *Kandelia obovata* and its role in response to the intertidal environment. Plant Cell Environ.

[37] Li J, Liu H, Yang C (2020). Genome-wide identification of *MYB* genes and expression analysis under different biotic and abiotic stresses in *Helianthus annuus* L. Ind Crop Prod.

[38] Wittkopp PJ, Kalay G (2012). *Cis*-regulatory elements: molecular mechanisms and evolutionary processes underlying divergence. Nat Rev Genet.

[39] Wu X, Ren Y, Jiang H (2021). Genome-wide identification and transcriptional expression analysis of annexin genes in *Capsicum annuum* and characterization of CaAnn9 in salt tolerance. Int J Mol Sci.

[40] He X, Liao L, Xie S (2020). Comprehensive analyses of the annexin (ANN) gene family in *Brassica rapa*, *Brassica oleracea* and *Brassica napus* reveals their roles in stress response. Sci Rep.

[41] Shin H, Brown RM (1999). GTPase activity and biochemical characterization of a recombinant cotton fiber annexin. Plant Physiol.

[42] Mcclung AD, Carroll AD, Battey NH (1994). Identification and characterization of ATPase activity associated with maize (*Zea mays*) annexins. Biochem J.

[43] Xia W, Yu H, Cao P (2017). Identification of TIFY family genes and analysis of their expression profiles in response to phytohormone treatments and *Melampsora laricipopulina* infection in poplar. Front Plant Sci.

[44] Zhang J, Yuan H, Li Y (2020). Genome sequencing and phylogenetic analysis of allotetraploid *Salix matsudana* Koidz. Hortic Res.

[45] Schilling S, Kennedy A, Pan S (2020). Genome-wide analysis of MIKC-type MADS-box genes in wheat: pervasive duplications, functional conservation and putative neofunctionalization. New Phytol.

[46] Wu Y, Wu S, Wang X (2022). Genome-wide identification and characterization of the bHLH gene family in an ornamental woody plant *Prunus mume*. Hortic Plant J.

[47] Chalhoub B, Denoeud F, Liu S (2014). Wincker, Early allopolyploid evolution in the post-Neolithic *Brassica napus* oilseed genome. Science.

[48] Zou J, Mao L, Qiu J (2019). Genome-wide selection footprints and deleterious variations in young Asian allotetraploid rapeseed. Plant Biotechnol J.

[49] Jakab G, Ton J, Flors V, L. et al. Enhancing Arabidopsis salt and drought stress tolerance by chemical priming for its abscisic acid responses. Plant Physiol. 2005;139:267–274. 10.1104/pp.105.065698 PMC1203376 16113213

[50] Ding P, Ding Y (2020). Stories of salicylic acid: a plant defense hormone. Trends Plant Sci.

[51] Wu X, J Yan, Wu Y, et al. Proteomic analysis by iTRAQ-PRM provides integrated insight into mechanisms of resistance in pepper to *Bemisia tabaci* (Gennadius). BMC Plant Biol. 2019;19:1–19. 10.1186/s12870-019-1849-0 PMC6588876 31226939

[52] Zhao J, Li L, Liu Q (2019). A MIF-like effector suppresses plant immunity and facilitates nematode parasitism by interacting with plant annexins. J Exp Bot.

[53] Gantait S, Rani Sinniah U, Ali N (2015). Gibberellins-a multifaceted hormone in plant growth regulatory network. Curr Protein Pept Sci.

[54] Wu X, Wang Y, Bian Y (2022). A critical review on plant annexin: Structure, function, and mechanism. Plant Physiol Biochem.

[55] Malgorzata L, Wojciech R, Karolina M (2018). Nucleus- and plastid-targeted annexin 5 promotes reproductive development in Arabidopsis and is essential for pollen and embryo formation. BMC Plant Biol.

[56] Kodavali PK, Dudkiewicz M, Pikuła S (2014). Bioinformatics analysis of bacterial annexins–putative ancestral relatives of eukaryotic annexins. PLoS ONE.

[57] Jiang P, Gao J, Mu J (2020). Interaction between serine carboxypeptidase-like protein TtGS5 and Annexin D1 in developing seeds of *Triticum timopheevi*. J Appl Genet.

[58] Teichmann T, Muhr M (2015). Shaping plant architecture. Front. Plant Sci.

[59] Ma L, Ye J, Yang Y (2019). The SOS2-SCaBP8 complex generates and fine-tunes an AtANN4-dependent calcium signature under salt stress. Dev Cell.

[60] Yadav D, Ahmed I, Kirti PB, Genome-wide identification and expression profiling of annexins in *Brassica rapa* and their phylogenetic sequence comparison with *B. juncea* and *A. thaliana* annexins. Plant Gene. 2015;4:109–124.

