# Supplementary material for: Comprehensive analysis of annexin gene family and its expression in response to branching architecture and salt stress in crape myrtle
Source: BMC Plant Biol. 2024 Jan 30;24:78. doi: 10.1186/s12870-024-04748-8 (PMC10826223; doi:10.1186/s12870-024-04748-8)
Supplement: Supplementary file 1 — Supplementary file1 (DOCX 4232 kb) [file 12870_2024_4748_MOESM1_ESM.docx]

| 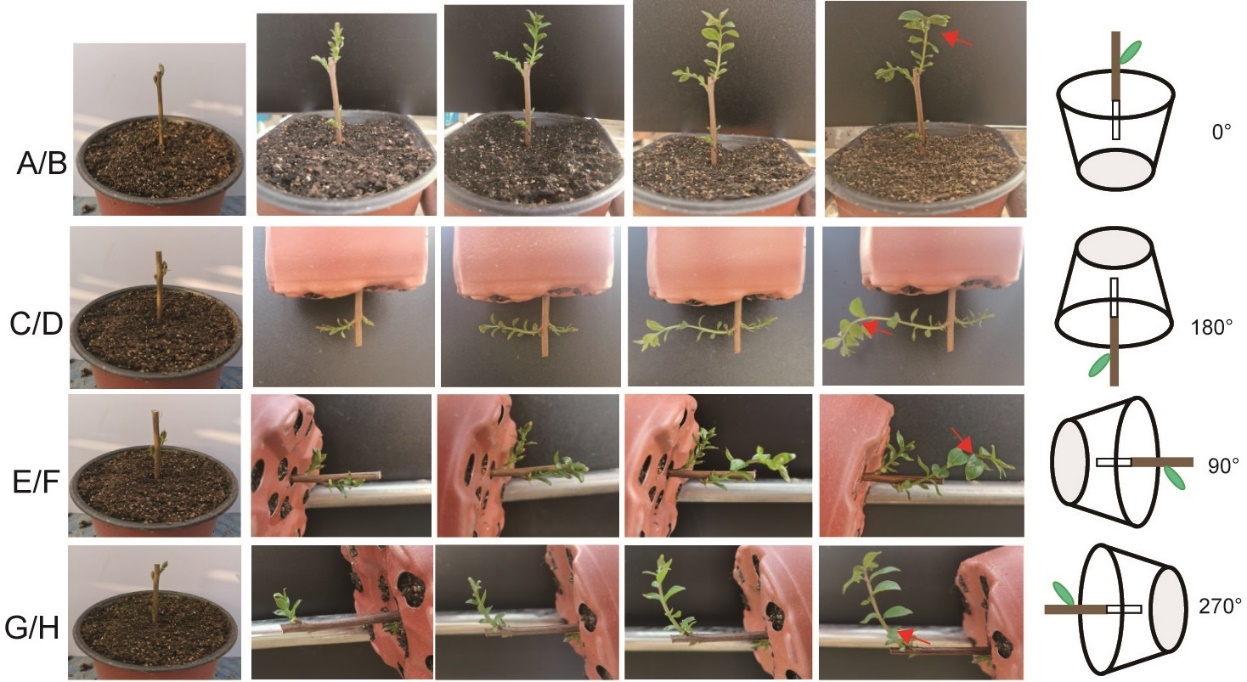 |
| --- |
| **Supplemental Fig. 1** Branch growth of *L. indica* under different rotation angle. A/B indicated *L. indica* maintaining normal growth; C/D indicated *L. indica* treated by rotation of 180°; E/F indicated *L. indica* treated by rotation of 90°; and G/H indicated *L. indica* treated by rotation of 270°. Among of them, the A, C, E, and G indicated the upper tissues of the bending sites (UTBS), while the B, D, F and H represented the lower tissues of the bending sites (LTBS). Bending of the branches were indicated by the red arrows. |

| 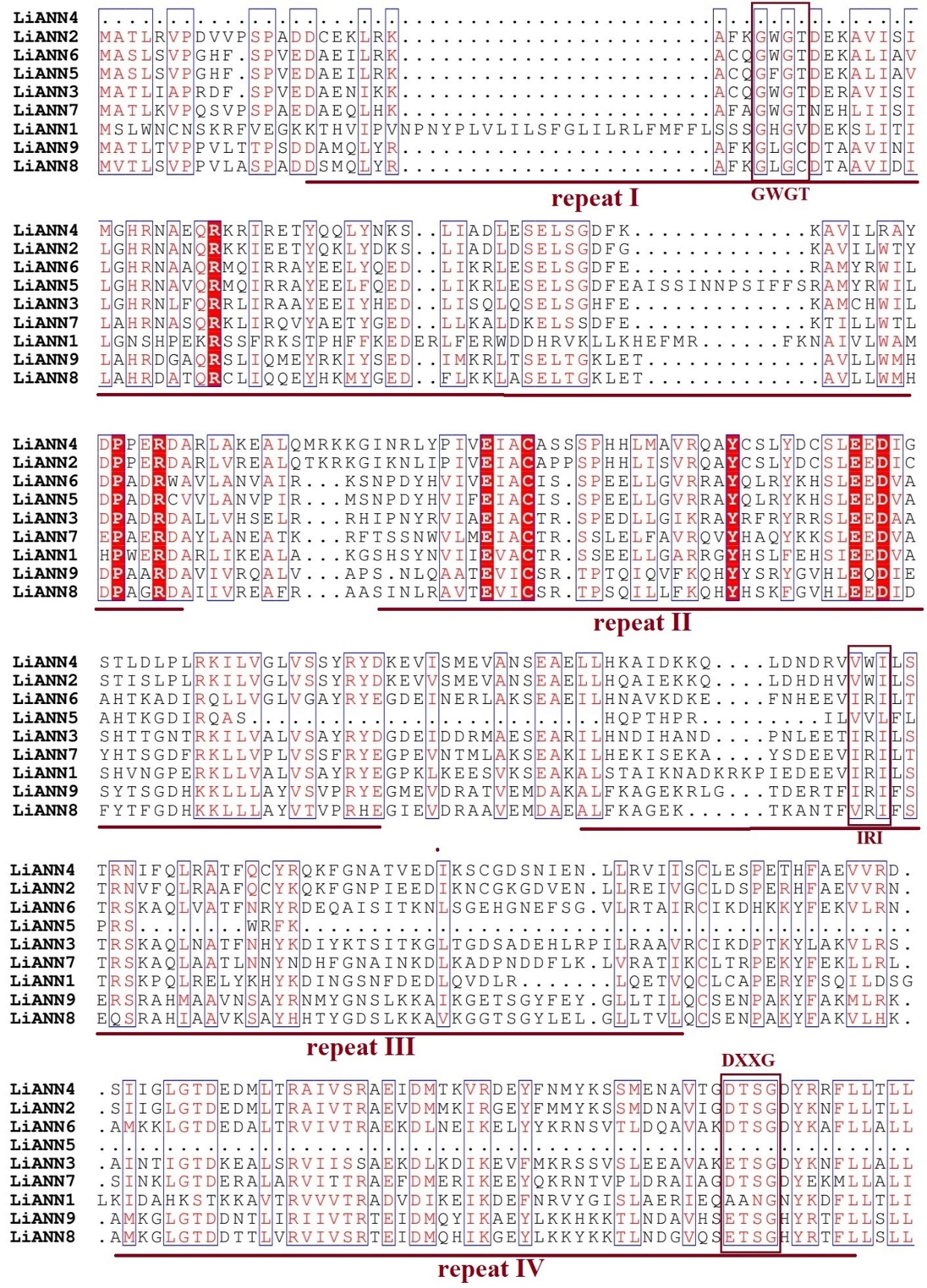 |
| --- |
| **Supplemental Fig. 2** Multiple alignment of the LiANN proteins. The conserved domains, including IRI, GWGT, and DXXG were remarked by red rectangles. |

| 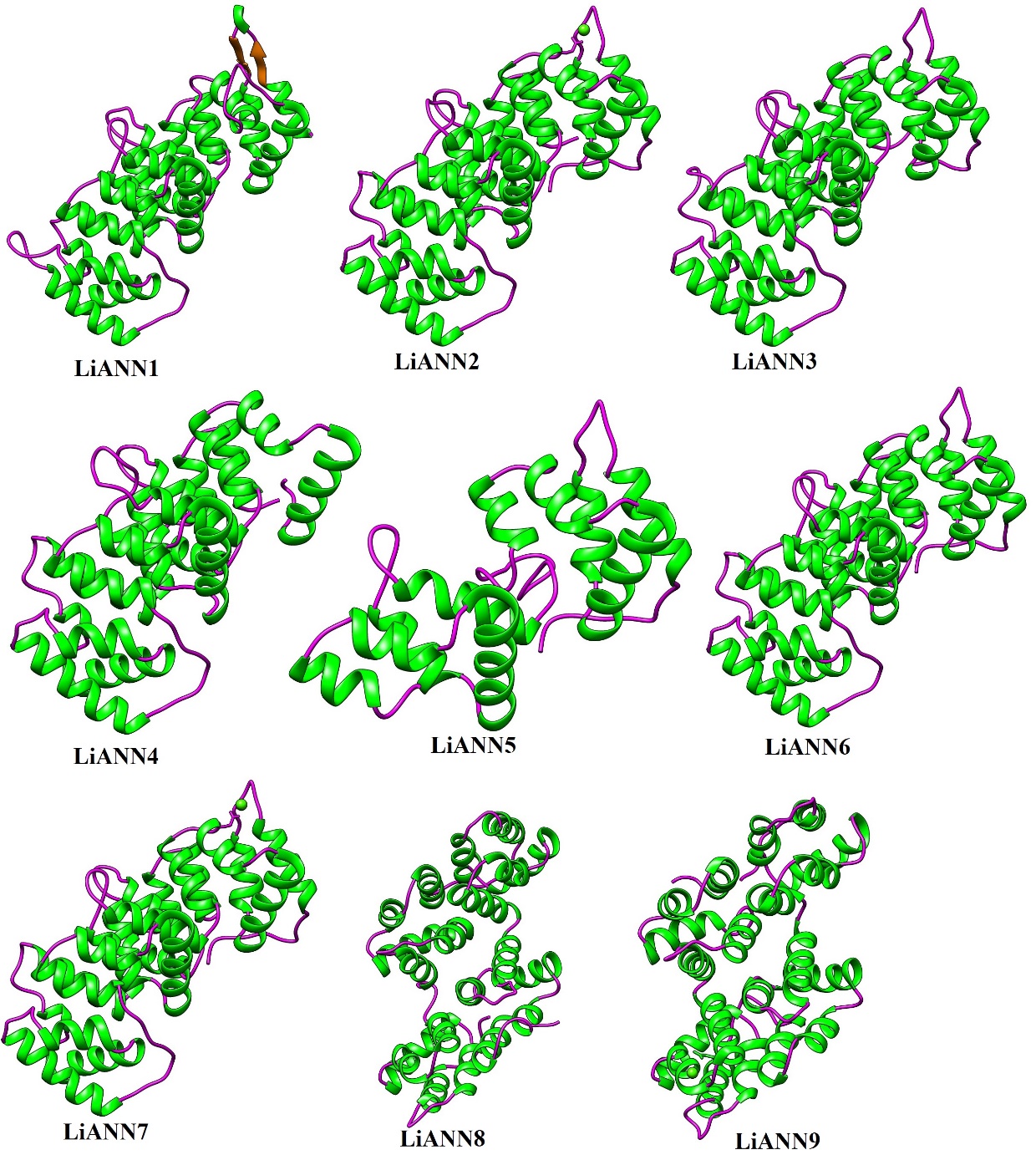 |
| --- |
| **Supplemental Fig. 3** The 3D structure analysis of the LiANN proteins. The coli, α-helix and strand are indicated in magenta, green, and orange, respectively. |

| 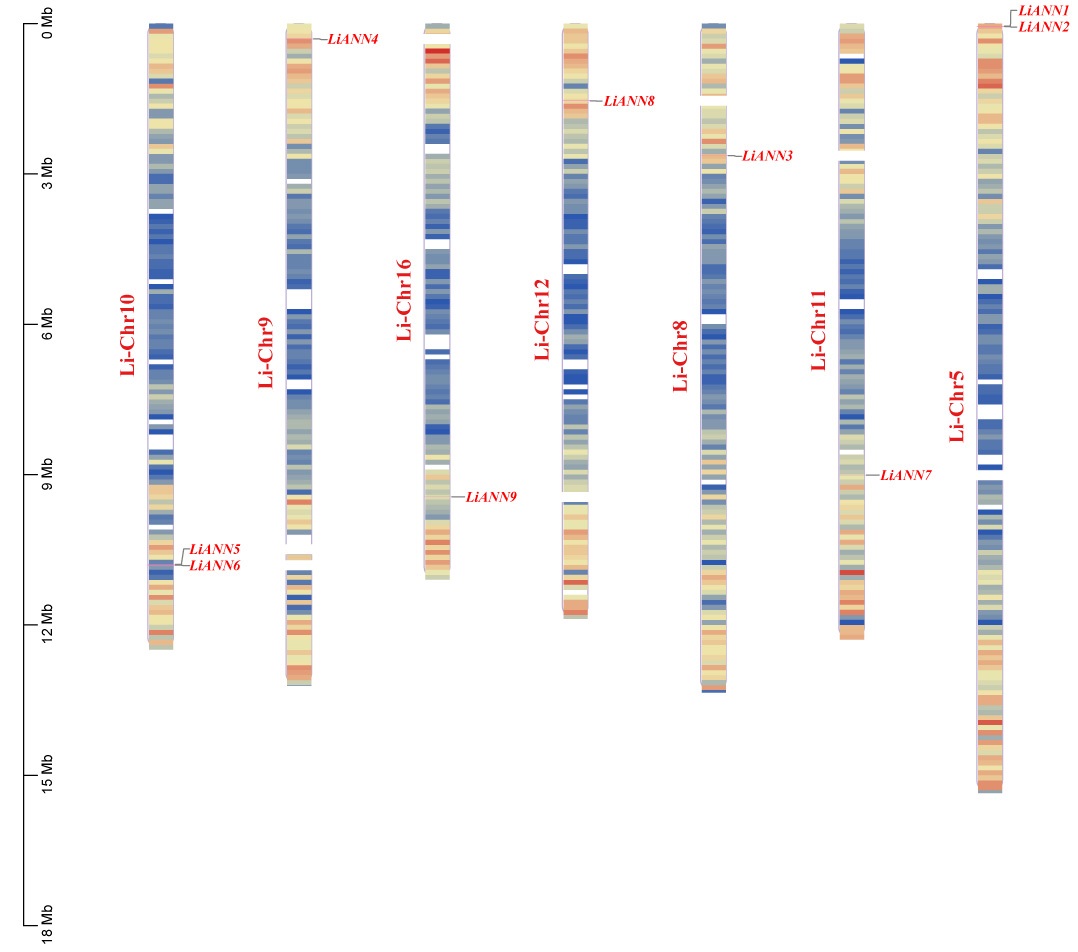 |
| --- |
| **Supplemental Fig. 4** Chromosomal distributions of the *LiANN* genes in crape myrtle. |

| 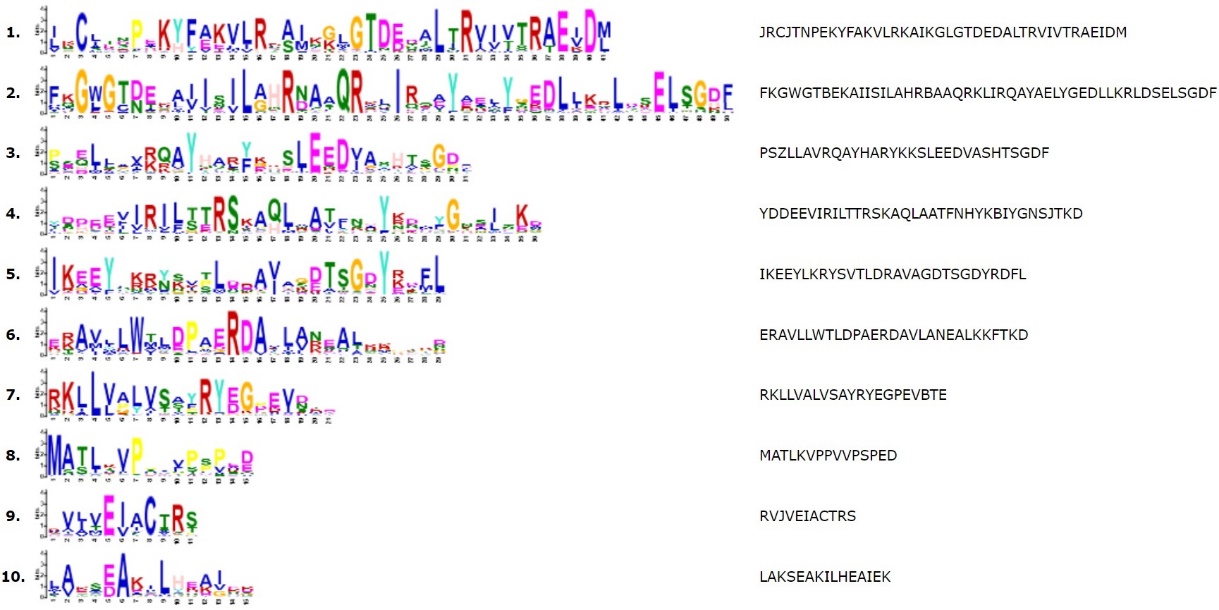 |
| --- |
| **Supplemental Fig.5** The logos of conserved motif patterns of the LiANNs |

| 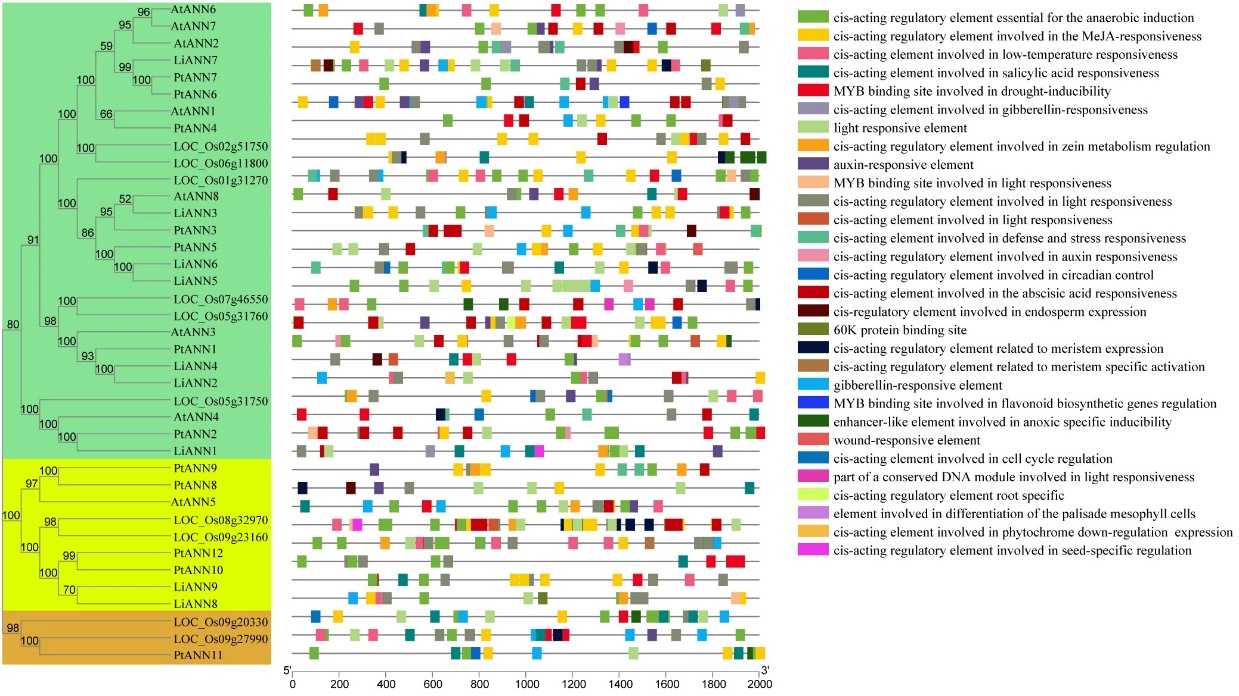 |
| --- |
| **Supplemental Fig. 6** Predicted *cis*-elements in the *LiANN* promoters. The *LiANN* promoter sequences (-2000 bp) were analyzed by PlantCARE. |

| 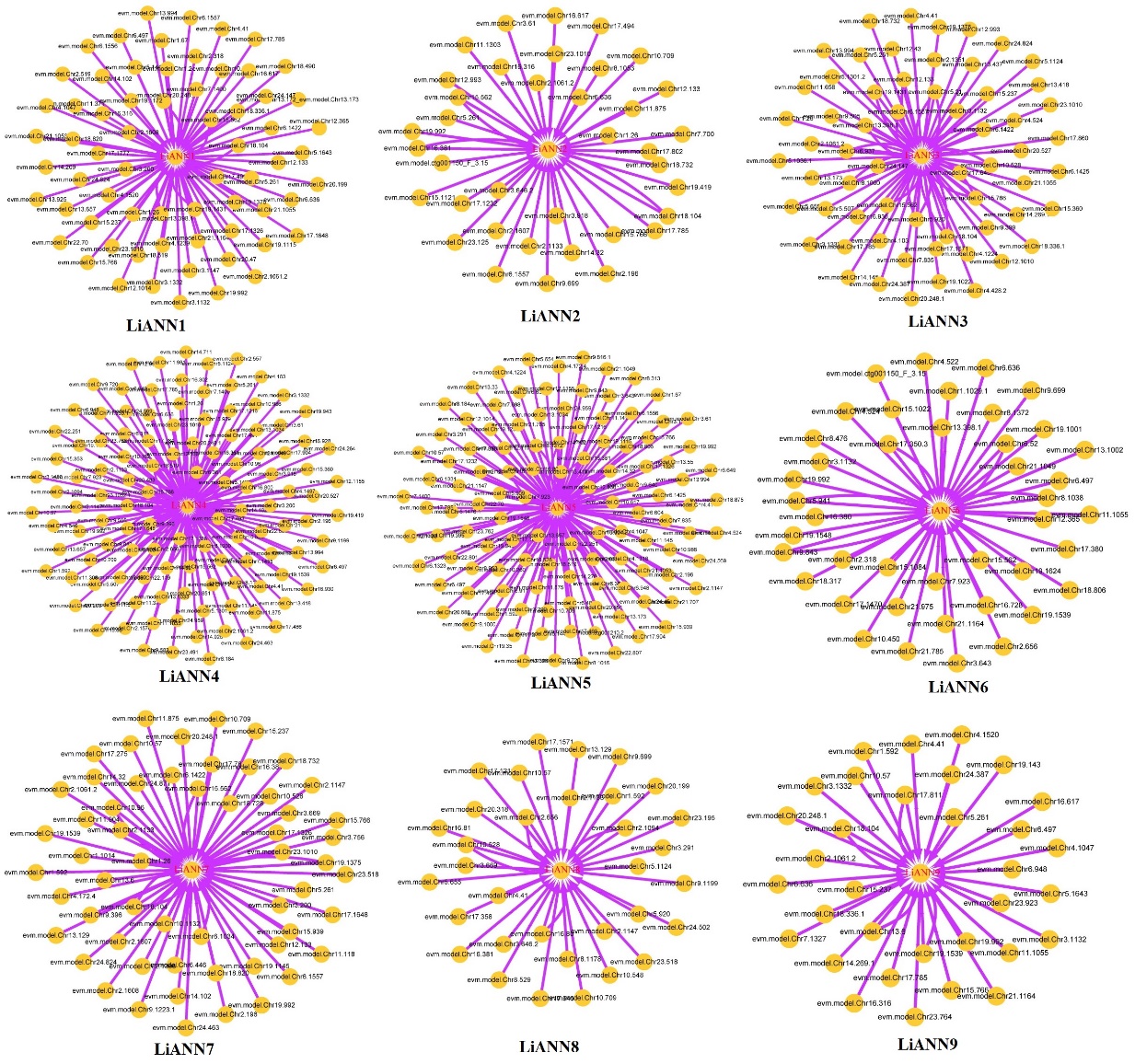 |
| --- |
| **Supplemental Fig. 7** The regulatory network between transcription factors (TFs) and *LiANN* genes. The purplish red lines highlighted putative regulatory relationship between TFs and *LiANN* genes. The putative TFs and *LiANN* genes were indicated by lavender circles and square, respectively. |

| 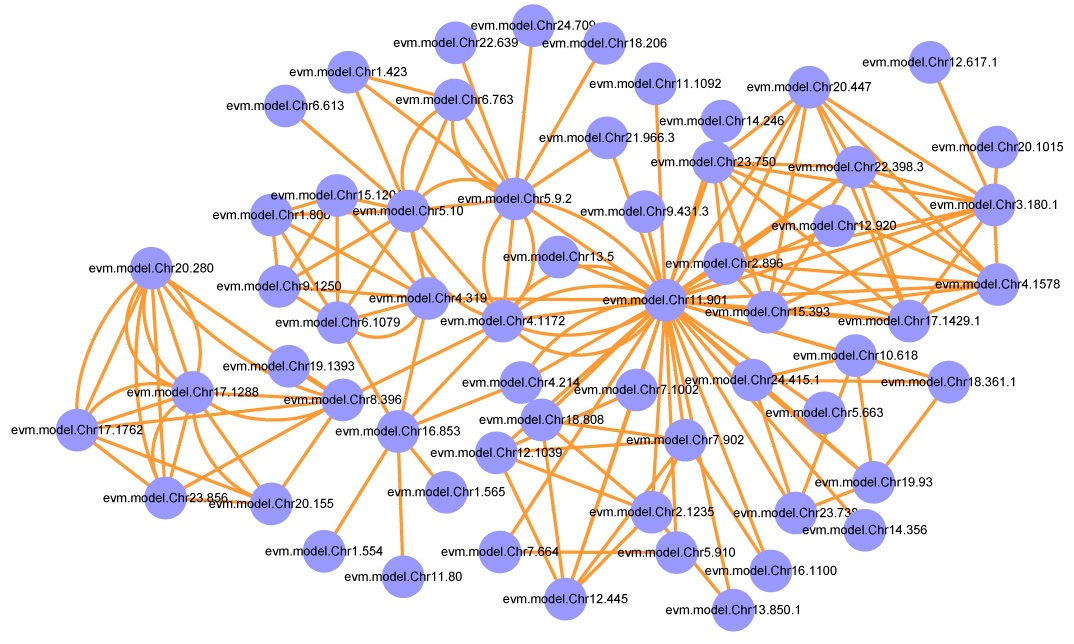 |
| --- |
| **Supplemental** **Fig. 8** The putative interaction network analysis of the LiANNs. The turquoise circles represented LiANNs and corresponding interaction proteins, and orange lines highlighted the putative interaction relationship. |

| 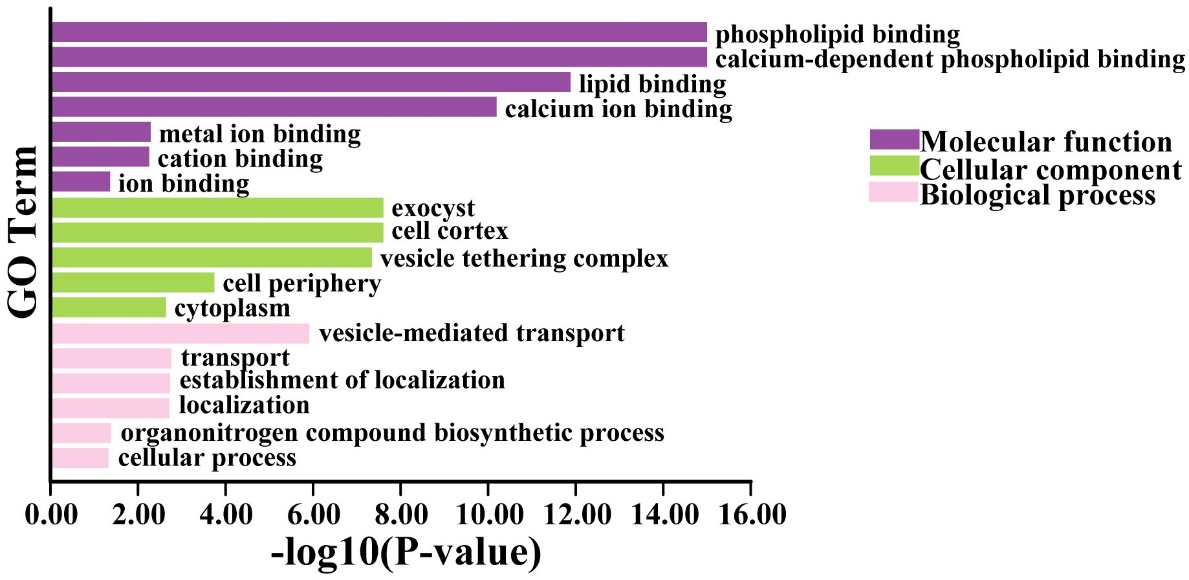 |
| --- |
| **Supplemental** **Fig. 9** Gene ontology (GO) enrichment analysis of the LiANNs from *L. indica*. |

| 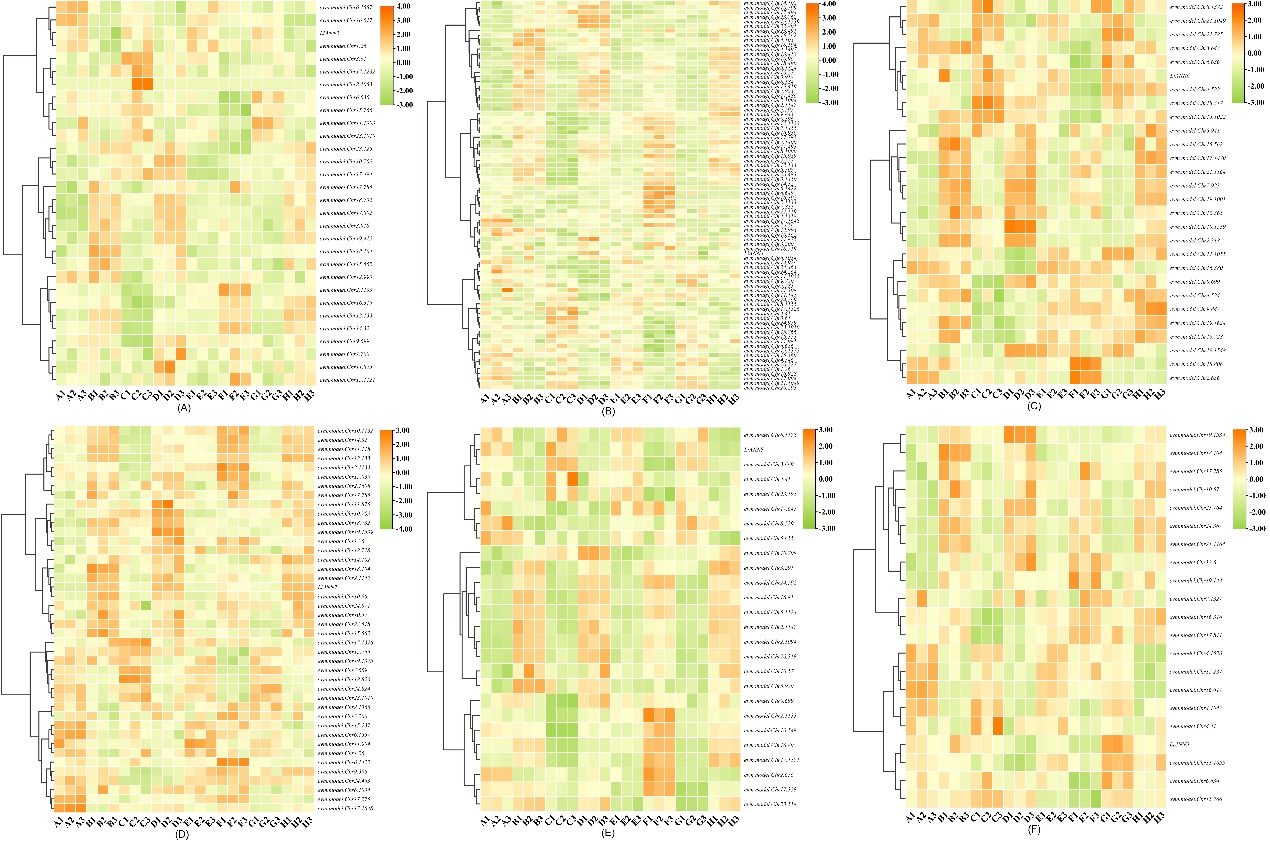 |
| --- |
| **Supplemental** **Fig. 10** Expression patterns of the differentially expressed *LiANNs*, including *LiANN2* (A), *LiANN4* (B), *LiANN6* (C), *LiAN7* (D), *LiANN8* (E), and *LiANN9* (F), and corresponding TF genes during the branching architecture of *L. indica*. Expression data were performed with log2 normalization. The color scale represented relative expression levels. A/B indicated *L. indica* maintaining normal growth; C/D indicated *L. indica* treated by rotation of 180°; E/F indicated *L. indica* treated by rotation of 90°; and G/H indicated *L. indica* treated by rotation of 270°. |

Supplemental tab. 1 The primers used in this study.

| Gene | Forward | Reverse |
| --- | --- | --- |
| *LiANN1* | CTGTGAAATCCGAGGCGAAG | GCACTGGACTGTTTCTTGCA |
| *LiANN2* | GCCGATGATTGCGAGAAACT | GTAAAGCCTCCCTCACCAGT |
| *LiANN3* | CGATGTGCCACTGGATCTTG | ACCAGCGCAACCAGAATTTT |
| *LiANN4* | TGTTGGATTGGTGAGCTCCT | TTACTGTCCCCGCAACTCTT |
| *LiANN5* | ATGGCTTCCCTCAGTGTTCC | AAGGCGCTTGATAAGGTCCT |
| *LiANN6* | CGGGTGAGCATGGAAATGAG | GGTGACACTGTTTCGCTTGT |
| *LiANN7* | GGTAAACACAATGCTGGCCA | TCGGCCTTCAGATCCTTGTT |
| *LiANN8* | TGAAGAAGGCAGTGAAGGGT | TGCTGCATGTCTATCTCGGT |
| *LiANN9* | GCCACCGAAGTCATCTGTTC | CAGCCTCTTCTCTCCTGCTT |
| *LiActin* | TGGTCTTGGAACAGTTGGAC | AACTCCACCATCCGTCTGA |

Supplemental tab. 2 Genome-wide analysis of *L. indica* *ANN* members based on the Pfam and alignment with AtANNs.

| pfam | alignment | Integration |
| --- | --- | --- |
| *evm.model.Chr10.925* | *evm.model.Chr10.927* | *evm.model.Chr10.927* |
| *evm.model.Chr10.927* | *evm.model.Chr11.901* | *evm.model.Chr11.901* |
| *evm.model.Chr11.901* | *evm.model.Chr12.256* | *evm.model.Chr12.256* |
| *evm.model.Chr12.256* | *evm.model.Chr16.853* | *evm.model.Chr16.853* |
| *evm.model.Chr16.853* | *evm.model.Chr5.10* | *evm.model.Chr5.10* |
| *evm.model.Chr3.528* | *evm.model.Chr5.9.2* | *evm.model.Chr5.9.2* |
| *evm.model.Chr5.10* | *evm.model.Chr8.396* | *evm.model.Chr8.396* |
| *evm.model.Chr5.9.2* | *evm.model.Chr9.51* | *evm.model.Chr9.51* |
| *evm.model.Chr6.1140* |  |  |
| *evm.model.Chr8.396* |  |  |
| *evm.model.Chr9.51* |  |  |

Supplemental tab. 3 The accession number of the *LiANN* genes.

| *A. thaliana* | | *O. sativa* | *L. indica* | |
| --- | --- | --- | --- | --- |
| Gene name | Accession number | Accession number | Gene name | Accession number |
| *AtANN2* | *AT5G65020* | LOC_Os09g23160.1 | *LiANN1* | *evm.model.Chr5.9.2* |
| *AtANN5* | *AT1G68090* | LOC_Os08g32970.1 | *LiANN2* | *evm.model.Chr5.10* |
| *AtANN1* | *AT1G35720* | LOC_Os06g11800.1 | *LiANN3* | *evm.model.Chr8.396* |
| *AtANN7* | *AT5G10230* | LOC_Os02g51750.1 | *LiANN4* | *evm.model.Chr9.51* |
| *AtANN6* | *AT5G10220* | LOC_Os05g31760.1 | *LiANN5* | *evm.model.Chr10.925* |
| *AtANN3* | *AT2G38760* | LOC_Os01g31270.1 | *LiANN6* | *evm.model.Chr10.927* |
| *AtANN8* | *AT5G12380* | LOC_Os07g46550.1 | *LiANN7* | *evm.model.Chr11.901* |
| *AtANN3* | *AT2G38750* | LOC_Os09g27990.1 | *LiANN8* | *evm.model.Chr12.256* |
|  |  | LOC_Os05g31750.1 | *LiANN9* | *evm.model.Chr16.853* |
|  |  | LOC_Os09g20330.1 |  |  |
|  |  |  |  |  |
|  |  |  |  |  |

Supplemental tab. 4 Characterization of the LiANN members.

| Gene name | Accession number | CDS length (bp) | Number of aa | Molecular weight | Theoretical PI | Instability index | Stability | Aliphatic index | Grand average  of hydropathicity  (GRAVY) | subcellular  localization |
| --- | --- | --- | --- | --- | --- | --- | --- | --- | --- | --- |
| *LiANN1* | *evm.model.Chr5.9.2* | 1044 | 347 | 39956.9 | 9.03 | 41.93 | unstable | 91.87 | -0.433 | Cytoplasm |
| *LiANN2* | *evm.model.Chr5.10* | 960 | 319 | 36040.5 | 5.89 | 39.88 | stable | 95.96 | -0.265 | Cytoplasm |
| *LiANN3* | *evm.model.Chr8.396* | 954 | 317 | 35876.8 | 6.61 | 54.85 | unstable | 94.89 | -0.418 | Cytoplasm |
| *LiANN4* | *evm.model.Chr9.51* | 849 | 282 | 32244.9 | 6.55 | 48.58 | unstable | 93.05 | -0.377 | Cytoplasm |
| *LiANN5* | *evm.model.Chr10.925* | 549 | 182 | 20875 | 7.06 | 61.04 | unstable | 94.34 | -0.214 | Cytoplasm |
| *LiANN6* | *evm.model.Chr10.927* | 942 | 313 | 35776.7 | 6.53 | 40.83 | unstable | 95.11 | -0.463 | Cytoplasm |
| *LiANN7* | *evm.model.Chr11.901* | 951 | 316 | 35955.9 | 6.29 | 37.24 | stable | 91.17 | -0.443 | Cytoplasm |
| *LiANN8* | *evm.model.Chr12.256* | 957 | 318 | 35568 | 8.49 | 40.72 | unstable | 92.96 | -0.188 | Cytoplasm |
| *LiANN9* | *evm.model.Chr16.853* | 948 | 315 | 35642.1 | 9.18 | 34.91 | stable | 87.08 | -0.318 | Cytoplasm |

Supplemental tab. 5 Phosphorylation of the LiANN members.

| LiANN1 | | | LiANN2 | | | LiANN3 | | | LiANN4 | | | LiANN5 | | | LiANN6 | | | LiANN7 | | | LiANN8 | | | LiANN9 | | |
| --- | --- | --- | --- | --- | --- | --- | --- | --- | --- | --- | --- | --- | --- | --- | --- | --- | --- | --- | --- | --- | --- | --- | --- | --- | --- | --- |
| # x (Site) | Score | Kinase | # x (Site) | Score | Kinase | # x (Site) | Score | Kinase | # x (Site) | Score | Kinase | # x (Site) | Score | Kinase | # x (Site) | Score | Kinase | # x (Site) | Score | Kinase | # x (Site) | Score | Kinase | # x (Site) | Score | Kinase |
| 2S | 0.65 | PKA | 12S | 0.99 | unsp | 11S | 1 | unsp | 23S | 0.58 | PKA | 5S | 0.612 | PKC | 3S | 0.519 | cdc2 | 3T | 0.87 | PKC | 5S | 0.58 | PKC | 3T | 0.56 | PKC |
| 46S | 0.52 | cdc2 | 12S | 0.6 | cdk5 | 11S | 0.5 | RSK | 30S | 0.62 | unsp | 11S | 0.856 | unsp | 5S | 0.612 | PKC | 3T | 0.72 | unsp | 5S | 0.53 | PKA | 5T | 0.73 | PKC |
| 48S | 0.56 | CKI | 12S | 0.52 | p38MAPK | 52Y | 0.52 | EGFR | 30S | 0.54 | CKII | 11S | 0.598 | CKII | 11S | 0.856 | unsp | 12S | 0.99 | unsp | 5S | 0.53 | cdc2 | 11T | 0.57 | unsp |
| 48S | 0.51 | cdc2 | 12S | 0.51 | GSK3 | 56Y | 0.96 | unsp | 33S | 0.55 | cdc2 | 28T | 0.503 | CKII | 11S | 0.598 | CKII | 12S | 0.56 | p38MAPK | 12S | 0.98 | unsp | 12T | 0.94 | unsp |
| 56S | 0.54 | CKI | 29T | 0.51 | CKII | 62S | 0.54 | ATM | 79S | 0.62 | PKC | 66S | 0.993 | unsp | 56Y | 0.95 | unsp | 12S | 0.52 | CKII | 12S | 0.61 | cdk5 | 12T | 0.59 | cdk5 |
| 64S | 0.97 | unsp | 53Y | 0.92 | unsp | 69S | 0.76 | unsp | 80S | 0.51 | cdc2 | 66S | 0.572 | RSK | 56Y | 0.505 | INSR | 44S | 0.98 | unsp | 12S | 0.52 | p38MAPK | 12T | 0.5 | p38MAPK |
| 64S | 0.57 | cdc2 | 53Y | 0.52 | SRC | 69S | 0.54 | cdc2 | 81S | 0.51 | cdk5 | 66S | 0.558 | PKA | 66S | 0.993 | unsp | 44S | 0.75 | PKC | 12S | 0.5 | GSK3 | 57Y | 0.51 | INSR |
| 70S | 0.57 | PKC | 60S | 0.58 | PKA | 113S | 1 | unsp | 94S | 0.92 | unsp | 69S | 0.599 | CKII | 66S | 0.575 | RSK | 44S | 0.62 | DNAPK | 44T | 0.97 | unsp | 58S | 0.52 | CKII |
| 71S | 1 | unsp | 67S | 0.76 | unsp | 131S | 1 | unsp | 96Y | 0.56 | unsp | 69S | 0.532 | cdc2 | 66S | 0.554 | PKA | 44S | 0.61 | RSK | 44T | 0.63 | PKC | 66T | 0.85 | unsp |
| 71S | 0.9 | PKC | 67S | 0.51 | CKII | 131S | 0.81 | PKA | 99S | 0.59 | CKII | 76S | 0.733 | unsp | 69S | 0.602 | unsp | 44S | 0.51 | ATM | 44T | 0.57 | DNAPK | 67S | 0.97 | unsp |
| 75S | 0.98 | unsp | 70S | 0.62 | unsp | 131S | 0.51 | CKII | 99S | 0.58 | unsp | 77S | 0.511 | cdc2 | 69S | 0.556 | CKII | 53Y | 0.51 | EGFR | 44T | 0.55 | ATM | 67S | 0.6 | RSK |
| 75S | 0.53 | PKG | 70S | 0.56 | cdc2 | 138S | 0.55 | CKI | 99S | 0.52 | cdc2 | 86S | 0.544 | PKA | 69S | 0.508 | cdc2 | 56T | 0.71 | unsp | 53Y | 0.56 | EGFR | 70T | 0.78 | unsp |
| 75S | 0.52 | PKC | 70S | 0.53 | CKI | 169S | 0.53 | CKII | 99S | 0.5 | DNAPK | 86S | 0.526 | PKG | 98S | 0.968 | unsp | 56T | 0.61 | CKII | 57Y | 0.53 | INSR | 70T | 0.54 | PKC |
| 76T | 0.74 | unsp | 70S | 0.51 | CKII | 188T | 0.8 | unsp | 106S | 0.86 | unsp | 111S | 0.937 | unsp | 98S | 0.701 | PKA | 57Y | 0.51 | unsp | 70T | 0.81 | unsp | 113T | 0.56 | p38MAPK |
| 76T | 0.54 | PKG | 81T | 0.61 | PKC | 193S | 0.99 | unsp | 106S | 0.52 | CKII | 111S | 0.657 | PKA | 98S | 0.534 | PKG | 70S | 0.57 | CKII | 100S | 0.76 | PKA | 113T | 0.5 | cdk5 |
| 76T | 0.52 | RSK | 98T | 0.97 | unsp | 193S | 0.85 | PKC | 107T | 0.54 | cdc2 | 115Y | 0.961 | unsp | 102Y | 0.971 | unsp | 70S | 0.52 | CKI | 116S | 0.75 | PKA | 115T | 0.58 | unsp |
| 132S | 0.54 | PKA | 98T | 0.9 | PKC | 193S | 0.53 | RSK | 122S | 0.83 | PKC | 125S | 0.635 | CKII | 112S | 0.613 | CKII | 70S | 0.52 | cdc2 | 116S | 0.58 | DNAPK | 115T | 0.52 | PKG |
| 132S | 0.51 | PKC | 118S | 0.56 | unsp | 194T | 0.87 | PKC | 131S | 0.81 | unsp | 126S | 0.993 | unsp | 113S | 0.993 | unsp | 71S | 0.94 | unsp | 116S | 0.53 | RSK | 138S | 0.94 | unsp |
| 134S | 0.54 | unsp | 118S | 0.54 | cdk5 | 203T | 0.64 | PKC | 131S | 0.5 | CKII | 126S | 0.529 | CKII | 113S | 0.529 | CKII | 71S | 0.68 | CKII | 116S | 0.52 | ATM | 138S | 0.52 | DNAPK |
| 146S | 0.89 | unsp | 118S | 0.52 | p38MAPK | 203T | 0.52 | cdc2 | 137S | 0.96 | unsp | 144S | 0.981 | unsp | 131S | 0.981 | unsp | 90Y | 0.87 | unsp | 125Y | 0.51 | EGFR | 139Y | 0.78 | unsp |
| 146S | 0.56 | PKA | 118S | 0.51 | GSK3 | 207Y | 0.77 | unsp | 137S | 0.64 | CKII | 144S | 0.551 | PKA | 131S | 0.551 | PKA | 90Y | 0.51 | INSR | 125Y | 0.51 | unsp | 139Y | 0.56 | INSR |
| 146S | 0.51 | CKII | 124S | 0.77 | PKC | 211Y | 0.92 | unsp | 161S | 0.64 | PKC | 144S | 0.507 | CKII | 131S | 0.507 | CKII | 96T | 0.65 | unsp | 127S | 0.58 | unsp | 140T | 0.5 | cdc2 |
| 147S | 0.98 | unsp | 124S | 0.6 | unsp | 211Y | 0.57 | INSR | 162T | 0.67 | PKC | 153T | 0.525 | PKC | 140T | 0.526 | PKC | 100T | 0.64 | PKA | 140Y | 0.58 | INSR | 141S | 0.93 | unsp |
| 147S | 0.64 | PKA | 131S | 0.92 | unsp | 213T | 0.73 | PKC | 171T | 0.5 | cdc2 | 161S | 0.962 | unsp | 169S | 0.963 | unsp | 101S | 0.76 | unsp | 141T | 0.7 | PKC | 141S | 0.53 | CKII |
| 147S | 0.53 | RSK | 133Y | 0.56 | unsp | 223S | 0.94 | unsp | 189S | 0.78 | unsp | 178S | 0.64 | PKC | 169S | 0.579 | CKII | 101S | 0.74 | PKA | 141T | 0.58 | CKI | 157Y | 0.85 | unsp |
| 159S | 0.99 | unsp | 136S | 0.6 | CKII | 223S | 0.59 | CKII | 193S | 0.96 | unsp |  |  |  | 193T | 0.984 | unsp | 102S | 0.53 | PKA | 154T | 0.68 | PKC | 184T | 0.68 | unsp |
| 159S | 0.57 | CKII | 136S | 0.58 | unsp | 243T | 0.6 | PKC | 193S | 0.52 | PKA |  |  |  | 193T | 0.861 | PKC | 114S | 0.6 | PKA | 182T | 0.58 | unsp | 194S | 0.91 | unsp |
| 159S | 0.5 | PKC | 136S | 0.5 | DNAPK | 252S | 0.84 | unsp | 193S | 0.5 | CKII |  |  |  | 194T | 0.825 | PKC | 114S | 0.53 | CKII | 186T | 0.51 | PKC | 194S | 0.53 | PKC |
| 164S | 1 | unsp | 144T | 0.54 | cdc2 | 252S | 0.64 | PKA | 204S | 0.56 | CKII |  |  |  | 196S | 0.635 | PKA | 115S | 0.92 | unsp | 206Y | 0.56 | EGFR | 194S | 0.5 | RSK |
| 164S | 0.62 | CKII | 146S | 0.5 | cdc2 | 252S | 0.55 | PKG | 208S | 0.99 | unsp |  |  |  | 203T | 0.596 | PKC | 115S | 0.76 | PKA | 213S | 0.78 | PKC | 208Y | 0.8 | unsp |
| 164S | 0.5 | cdc2 | 159S | 0.83 | PKC | 256T | 0.55 | CKI | 208S | 0.52 | cdk5 |  |  |  | 214S | 0.502 | cdc2 | 115S | 0.56 | DNAPK | 223S | 0.71 | PKA | 208Y | 0.6 | EGFR |
| 171S | 0.87 | unsp | 168S | 0.88 | unsp | 259T | 0.97 | unsp | 220S | 0.98 | unsp |  |  |  | 220S | 0.972 | unsp | 125Y | 0.54 | EGFR | 225Y | 0.56 | unsp | 212Y | 0.52 | INSR |
| 199S | 0.99 | unsp | 168S | 0.51 | CKII | 259T | 0.72 | PKC | 220S | 0.77 | PKA |  |  |  | 228S | 0.538 | cdc2 | 132S | 0.99 | unsp | 232T | 0.63 | PKC | 215S | 0.83 | PKC |
| 202S | 0.68 | unsp | 174S | 0.96 | unsp | 259T | 0.55 | CKII | 220S | 0.6 | CKI |  |  |  | 269T | 0.557 | CKII | 139Y | 0.93 | unsp | 237S | 0.51 | cdc2 | 224T | 0.57 | PKC |
| 209T | 0.85 | PKC | 174S | 0.64 | CKII | 270S | 0.65 | unsp | 226T | 0.56 | CKII |  |  |  | 269T | 0.525 | PKC | 142S | 0.53 | cdc2 | 257T | 0.5 | CKII | 225S | 0.93 | unsp |
| 230S | 0.91 | unsp | 198S | 0.61 | PKC | 270S | 0.59 | CKII | 237S | 0.95 | unsp |  |  |  | 283Y | 0.926 | unsp | 142S | 0.51 | CKII | 261T | 0.55 | cdc2 | 225S | 0.53 | CKII |
| 230S | 0.91 | PKC | 199T | 0.79 | PKC | 271S | 0.77 | unsp | 237S | 0.57 | CKII |  |  |  | 287S | 0.922 | unsp | 155S | 0.72 | PKC | 268S | 0.89 | unsp | 227Y | 0.82 | unsp |
| 230S | 0.52 | RSK | 245S | 0.98 | unsp | 287S | 0.68 | PKA | 244T | 0.55 | unsp |  |  |  | 287S | 0.833 | PKA | 158Y | 0.73 | unsp | 268S | 0.53 | CKII | 234T | 0.61 | PKC |
| 231T | 0.87 | PKC | 245S | 0.55 | p38MAPK | 287S | 0.5 | PKG | 250Y | 0.93 | unsp |  |  |  | 298T | 0.809 | unsp | 170S | 0.64 | unsp | 268S | 0.51 | PKG | 239S | 0.52 | cdc2 |
| 231T | 0.51 | cdc2 | 257S | 0.94 | unsp | 288S | 0.99 | unsp | 250Y | 0.5 | SRC |  |  |  | 298T | 0.617 | PKC | 180S | 0.93 | unsp | 281Y | 0.51 | unsp | 259T | 0.53 | CKII |
| 233S | 0.63 | unsp | 257S | 0.8 | PKA | 288S | 0.83 | PKA | 250Y | 0.5 | INSR |  |  |  | 302Y | 0.561 | unsp | 180S | 0.72 | PKC | 281Y | 0.5 | EGFR | 270T | 0.54 | CKII |
| 250S | 0.95 | unsp | 257S | 0.61 | CKI | 288S | 0.52 | cdc2 | 256S | 0.95 | unsp |  |  |  | 302Y | 0.524 | INSR | 184Y | 0.95 | unsp | 288T | 0.59 | PKA | 278Y | 0.57 | INSR |
| 250S | 0.56 | CKII | 263T | 0.56 | CKII | 290S | 1 | unsp | 256S | 0.58 | CKII |  |  |  |  |  |  | 184Y | 0.51 | SRC | 295S | 0.51 | CKII | 290T | 0.58 | PKA |
| 250S | 0.54 | PKA | 274T | 0.73 | unsp | 299T | 0.72 | PKC | 257S | 0.74 | unsp |  |  |  |  |  |  | 185S | 1 | unsp | 297T | 0.55 | cdc2 | 290T | 0.52 | PKG |
| 277S | 0.9 | unsp | 274T | 0.56 | CKII | 299T | 0.57 | unsp | 263T | 0.52 | CKII |  |  |  |  |  |  | 185S | 0.65 | CKII |  |  |  | 297S | 0.63 | unsp |
| 277S | 0.65 | ATM | 293S | 0.95 | unsp | 299T | 0.53 | CKI | 266T | 0.8 | unsp |  |  |  |  |  |  | 194T | 0.98 | unsp |  |  |  | 300S | 0.53 | PKC |
| 282S | 0.56 | cdc2 | 303T | 0.55 | unsp | 299T | 0.51 | PKG | 276T | 0.86 | PKC |  |  |  |  |  |  | 194T | 0.86 | PKC |  |  |  |  |  |  |
| 291S | 0.99 | unsp | 303T | 0.52 | PKC | 303Y | 0.91 | unsp |  |  |  |  |  |  |  |  |  | 195T | 0.83 | PKC |  |  |  |  |  |  |
| 291S | 0.84 | PKC | 304S | 0.53 | CKII | 303Y | 0.55 | INSR |  |  |  |  |  |  |  |  |  | 197S | 0.68 | PKA |  |  |  |  |  |  |
| 292T | 0.88 | PKC | 307Y | 0.91 | unsp |  |  |  |  |  |  |  |  |  |  |  |  | 204T | 0.53 | cdc2 |  |  |  |  |  |  |
| 292T | 0.61 | unsp | 307Y | 0.54 | INSR |  |  |  |  |  |  |  |  |  |  |  |  | 235T | 0.75 | unsp |  |  |  |  |  |  |
| 297T | 0.53 | PKC | 313T | 0.81 | PKC |  |  |  |  |  |  |  |  |  |  |  |  | 235T | 0.75 | PKA |  |  |  |  |  |  |
| 322S | 0.66 | PKA |  |  |  |  |  |  |  |  |  |  |  |  |  |  |  | 235T | 0.73 | PKC |  |  |  |  |  |  |
| 322S | 0.55 | CKII |  |  |  |  |  |  |  |  |  |  |  |  |  |  |  | 240T | 0.67 | PKC |  |  |  |  |  |  |
| 335Y | 0.85 | unsp |  |  |  |  |  |  |  |  |  |  |  |  |  |  |  | 245Y | 0.8 | unsp |  |  |  |  |  |  |
| 341T | 0.89 | PKC |  |  |  |  |  |  |  |  |  |  |  |  |  |  |  | 253S | 0.82 | PKA |  |  |  |  |  |  |
|  |  |  |  |  |  |  |  |  |  |  |  |  |  |  |  |  |  | 253S | 0.67 | unsp |  |  |  |  |  |  |
|  |  |  |  |  |  |  |  |  |  |  |  |  |  |  |  |  |  | 259T | 0.52 | cdc2 |  |  |  |  |  |  |
|  |  |  |  |  |  |  |  |  |  |  |  |  |  |  |  |  |  | 269T | 0.95 | unsp |  |  |  |  |  |  |
|  |  |  |  |  |  |  |  |  |  |  |  |  |  |  |  |  |  | 270T | 0.84 | unsp |  |  |  |  |  |  |
|  |  |  |  |  |  |  |  |  |  |  |  |  |  |  |  |  |  | 270T | 0.56 | PKG |  |  |  |  |  |  |
|  |  |  |  |  |  |  |  |  |  |  |  |  |  |  |  |  |  | 270T | 0.53 | CKII |  |  |  |  |  |  |
|  |  |  |  |  |  |  |  |  |  |  |  |  |  |  |  |  |  | 283Y | 0.82 | unsp |  |  |  |  |  |  |
|  |  |  |  |  |  |  |  |  |  |  |  |  |  |  |  |  |  | 283Y | 0.54 | EGFR |  |  |  |  |  |  |
|  |  |  |  |  |  |  |  |  |  |  |  |  |  |  |  |  |  | 288T | 0.63 | PKA |  |  |  |  |  |  |
|  |  |  |  |  |  |  |  |  |  |  |  |  |  |  |  |  |  | 299T | 0.86 | unsp |  |  |  |  |  |  |
|  |  |  |  |  |  |  |  |  |  |  |  |  |  |  |  |  |  | 300S | 0.7 | unsp |  |  |  |  |  |  |
|  |  |  |  |  |  |  |  |  |  |  |  |  |  |  |  |  |  | 300S | 0.63 | CKII |  |  |  |  |  |  |
|  |  |  |  |  |  |  |  |  |  |  |  |  |  |  |  |  |  | 303Y | 0.91 | unsp |  |  |  |  |  |  |
|  |  |  |  |  |  |  |  |  |  |  |  |  |  |  |  |  |  | 303Y | 0.54 | INSR |  |  |  |  |  |  |
